# Probiotics: their action against pathogens can be turned around

**DOI:** 10.1038/s41598-021-91542-3

**Published:** 2021-06-24

**Authors:** Lian Gan, Wei-Hua Xu, Yuanyan Xiong, Zhaolin Lv, Jianwei Zheng, Yu Zhang, Jianhao Lin, Jingshu Liu, Shijun Chen, Mengqiu Chen, Qingqi Guo, Junfeng Wu, Jingjie Chen, Zhenhua Su, Jijia Sun, Yuhui He, Chuanhe Liu, Weifang Wang, Willy Verstraete, Patrick Sorgeloos, Tom Defoirdt, Qiwei Qin, Yiying Liu

**Affiliations:** 1 Joint Laboratory of Guangdong Province and Hong Kong Region on Marine Bioresource Conservation and Exploitation, College of Marine Sciences, South China Agricultural University, Guangzhou, 510642 China; 2 Guangdong Laboratory of Lingnan Modern Agricultural Science and Technology, Guangzhou, 510642 China; 3 MOE Key Laboratory of Gene Function and Regulation, State Key Laboratory of Biocontrol, School of Life Sciences, Sun Yat-Sen University, Guangzhou, 510275 China; 4 Instrumental Analysis and Research Center, South China Agricultural University, Guangzhou, 510642 China; 5 Guangzhou Customs Technology Center, Guangzhou, 510623 China; 6 Center for Microbial Ecology and Technology (CMET), Ghent University, 9000 Gent, Belgium; 7 Laboratory of Aquaculture and Artemia Reference Center, Faculty of Bioscience Engineering, Ghent University, 9000 Gent, Belgium

**Keywords:** Microbial communities, Microbial ecology, Bacterial genomics, Bacteria, Bacterial pathogenesis, Microbiology, Pathogens, Metabolomics

## Abstract

Probiotics when applied in complex evolving (micro-)ecosystems, might be selectively beneficial or detrimental to pathogens when their prophylactic efficacies are prone to ambient interactions. Here, we document a counter-intuitive phenomenon that probiotic-treated zebrafish (*Danio rerio*) were respectively healthy at higher but succumbed at lower level of challenge with a pathogenic *Vibrio* isolate. This was confirmed by prominent dissimilarities in fish survival and histology. Based upon the profiling of the zebrafish microbiome, and the probiotic and the pathogen shared gene orthogroups (genetic niche overlaps in genomes), this consequently might have modified the probiotic metabolome as well as the virulence of the pathogen. Although it did not reshuffle the architecture of the commensal microbiome of the vertebrate host, it might have altered the probiotic-pathogen inter-genus and intra-species communications. Such in-depth analyses are needed to avoid counteractive phenomena of probiotics and to optimise their efficacies to magnify human and animal well-being. Moreover, such studies will be valuable to improve the relevant guidelines published by organisations such as FAO, OIE and WHO.

## Introduction

Probiotics are defined as ‘Live microorganisms which when administered in adequate amounts confer a health benefit on the host’^1^. These microorganisms, including a number of lactic acid bacteria (LAB), have been increasingly applied in the domains of human medicine, food and agriculture production, etc.^2–13^. Contradictorily, a few studies have demonstrated that probiotics are not universally good as they may even provoke the virulence of the pathogens^14,15^.

In accordance with the conventional medicinal notion, under the treatment with the same dosage of a probiotic, a host confronting a higher and lower amount of pathogen tends to be more and less compromised, respectively. However, in practice, as probiotics are surrounded by dynamic vulnerable (micro-)ecosystems affected significantly by versatile biotic, abiotic, spatial, temporal and anthropogenic backgrounds, the potential antagonism of probiotics agaist pathogens might be subject to change.

**Figure 1:**
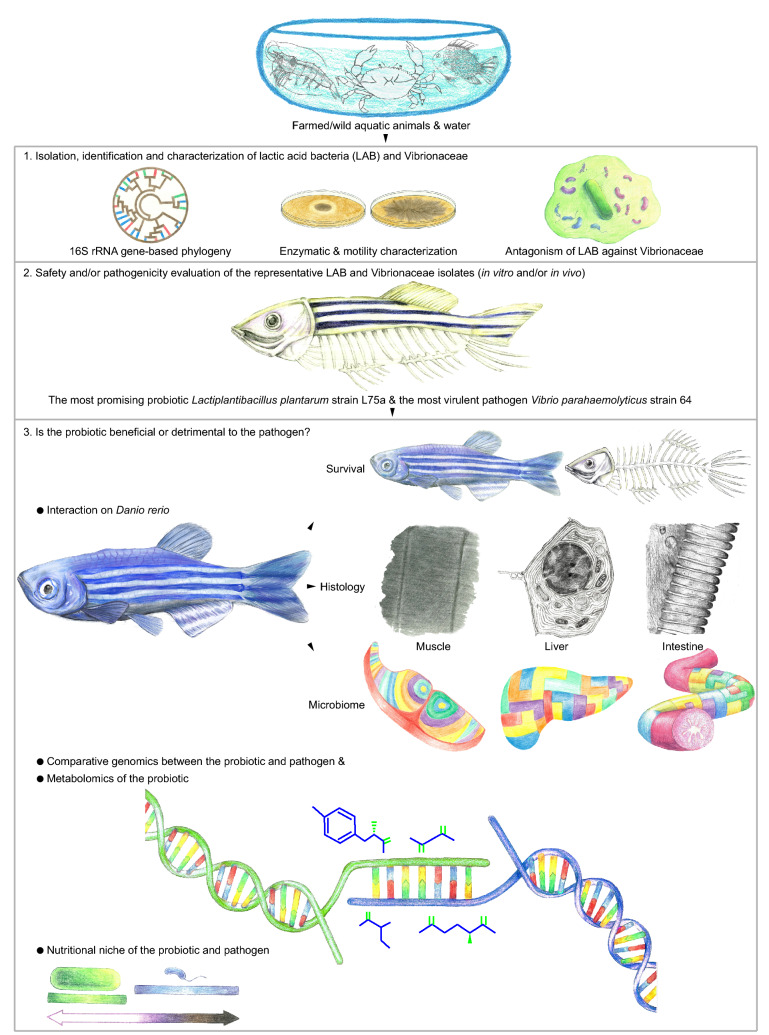
Overall strategy to decipher the beneficial or detrimental impacts of the probiotic against pathogen on zebrafish *D. rerio*, an aquatic animal model.

To decipher the influence of a probiotic against various loads of a pathogen on a vertebrate host, so as to steer and galvanize its beneficial efficacies, typical probiotic and pathogenic isolates belonging to well-known groups of microorganisms, i.e. LAB and Vibrionaceae, respectively, were used to establish an indicative interaction model in the aquatic animal model with zebrafish (*Danio rerio*) (Fig. 1). The outcome, exposing the selective contribution (dual contrary-antagonism) of probiotic and contradicting the aforementioned conventional medicinal notion, might launch a novel research theme to probe the impacts of probiotics on the pathogens during their bilateral communication.

## Results and discussion

### The probiotic is selectively beneficial or detrimental to the pathogen

As LAB are commonly affiliated with probiotics and Vibrionaceae with pathogens, our study started with the isolation of these two groups of bacteria from aquatic animals and waters (Fig. 1, Extended Data Table 1, Extended Data Fig. 1, detailed description of isolation, identification, characterization and selection of bacteria is shown in Supplementary information). After a series of screening and testing (Extended Data Figs. 2–6, Extended Data Table 2), the most promising probiotic isolate *Lactiplantibacillus plantarum* strain L75a and the most virulent isolate *Vibrio parahaemolyticus* strain 64 were exploited to study the impact of the probiotic on the pathogen: beneficial or detrimental (Fig. 1).

**Figure 2:**
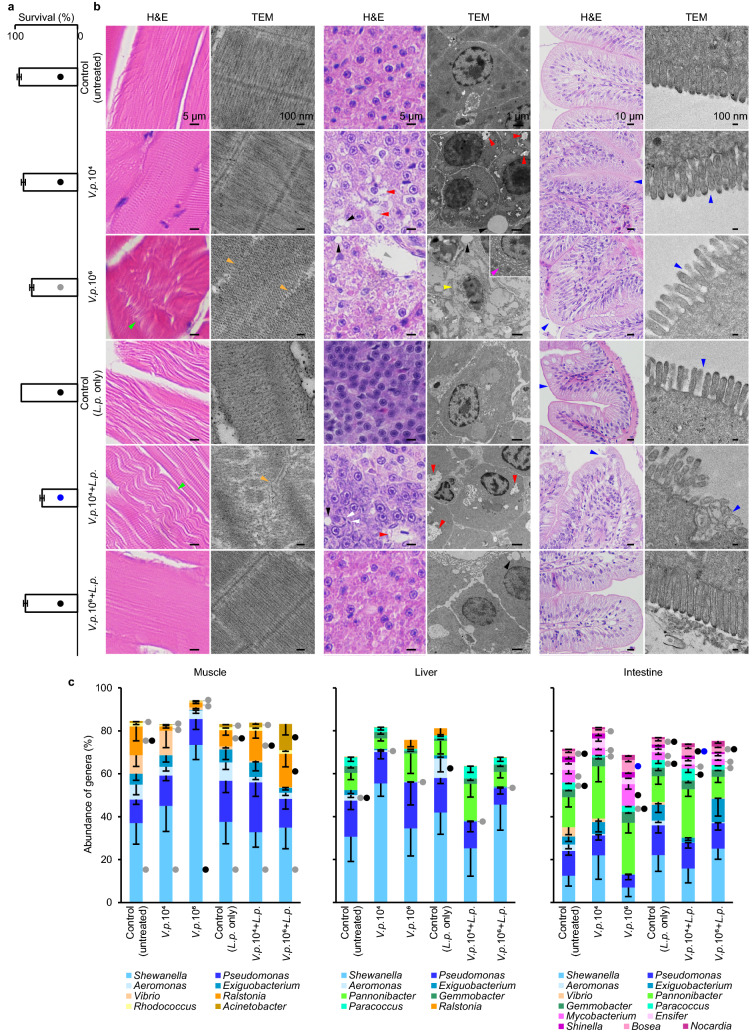
Impact of *Lactip. plantarum* strain L75a (*L.p.*) against *V. parahaemolyticus* strain 64 (*V.p.*) on the survival, histology and microbial community of *D. rerio*. The *D. rerio* were co-incubated with a lower or higher density of *V.p.* (10^4^ or 10^6^ cells ml^−1^, respectively) and supplemented with or without the fermented broth of *L.p.*. The control treatments were supplemented without (untreated) or with the fermented broth (*L.p.* only). Scoring and sample collection were conducted after 9 days of cultivation when the mean survival of at least 2 replicates of the same treatment just plunged to 50%. (**a**) Mean survival percentage of *D. rerio*. (**b**) Histological morphologies of the muscle, liver and intestine of *D. rerio* based on H&E-staining and TEM photomicrography. Green, orange, black, red, grey, white, yellow, pink and blue arrows indicate acute muscle necrosis (wavy shape), disappearing muscle fibres, lipid droplets, liver necrosis, liver parenchyma lysed area, (curved) rod-shaped bacterial-sized cells, disappearing liver cell membrane, swelling ribosomes, and damaged/malformed intestinal structure, respectively. (**c**) A total of 3367 operational taxonomic units (OTUs) were detected based on 16S rRNA gene amplicon sequencing, and the abundance percentage of the genera detected in the muscle, liver and intestine of *D. rerio* are shown. The detected genera without designated genus names and with mean percentage lower than 3.0% are not shown. (**a**) and (**c**), error bars represent S.E.M. (*N* = 3). In the same category, survival percentage of *D. rerio* (**a**) or abundance percentage of each genus (**c**), treatments with filled circles of different colours (black, grey or blue) indicate statistically significant differences.

Our research unveiled a phenomenon colliding with the prevailing pharmaceutical cognition: the *D. rerio* prescribed by a fixed dose of fermented broth of *Lactip. plantarum* strain L75a and treated with the higher and lower densities of *V. parahaemolyticus* strain 64 were respectively healthy and diseased/deceased—the survival percentage of *D. rerio* in the former treatment was significantly higher than that in the latter one (Fig. 2a); and commensurately, myolysis, ruptured muscle fibre, liver necrotic lesions and intestinal microvilli cracks were absent in the former treatment and present in the latter one (Fig. 2b). In contrast, the *D. rerio* that only received higher and lower densities of *V. parahaemolyticus* (without *Lactip. plantarum* inclusion) were severely and slightly succumbed, respectively (Fig. 2a,b). Such remarkable disparities in the fish survival and histology failed to be directly and solely related to the detected microbial compositions as there were no crucial discrepancies between the two treatments (*Lactip. plantarum* accompanying higher and lower densities of *V. parahaemolyticus*) in the muscle, liver and intestine of the host (Fig. 2c).

Although *Lactip. plantarum* strain L75a, alike other specific probiotic strains, is in compliance with the basic requisition as a probiotic (according to the safety evaluation and its antagonism against pathogens (Extended Data Figs. 2c, 3, 4, 5a,b and 6, Extended Data Table 2)), it should be applied rationally under specific physical, chemical and biological backgrounds of the host at specific disease stages, to optimize its prophylactic efficacy. Indeed, vast studies have demonstrated the beneficial efficacies of probiotics, including balancing microbial community and antagonizing pathogens^13,16^; in parallel, a few research hold the opposite opinions that, probiotics are capable of enhancing the virulence of bacterial pathogens^14,15^. Interestingly, our study even discovers a novel phenomenon that the probiotic is selectively beneficial or detrimental to the pathogen, and it offers an important starting point to develop targeted probiotic treatments based on the pathogen density.

### Are the probiotic-pathogen shared gene orthogroups attributed to the benefit or detriment of the probiotic to the pathogen?

Genome analyses of *Lactip. plantarum* strain L75a, *V. parahaemolyticus* strain 64, and 191 LAB and 225 Vibrionaceae reference strains disclose 47 and 91 shared gene orthogroups of single- and multiple-copy, respectively; meanwhile, the genomes of *Lactip. plantarum* strain L75a and *V. parahaemolyticus* strain 64 contain 271 and 583 shared gene orthogroups of single- and multiple-copy, respectively. After exclusion of those related to regular basic biological processes, the sole remaining shared gene orthogroup is related to quorum sensing and represented in both *Lactiplantibacillus*^17^ and *Vibrio* (Table 1). The corresponding gene is *luxS*, which is responsible for the production and detection of the signal molecule autoinducer-2 in quorum sensing circuit^19^. According to Song *et al.*^15,17^, *Lactiplantibacillus* and *Vibrio* are capable of using the signal molecules (autoinducers) to launch inter-genus communication via a quorum-sensing cascade.

**Table 1:** The probiotic-pathogen shared gene orthogroups (genetic niche overlap) that might rationalize the benefit or detriment of the probiotic to the pathogen.

Strain	Copy number	Gene ontology [number]	Protein name [gene name]	Organism [protein identity]
*Lactip. plantarum* strain L75a & *V. parahaemolyticus* strain 64	Single	Quorum sensing [0009372]	Autoinducer-2 production protein LuxS [*luxS*]	*Lactip. plantarum* ATCC BAA-793 [100%], *V. parahaemolyticus* O3:K6 strain RIMD 2210633 [99.419%]
	Multiple	–	–	–
*Lactip. plantarum* strain L75a, *V. parahaemolyticus* strain 64, 191 LAB & 225 Vibrionaceae reference strains	Single	–	–	–
	Multiple	–	–	–

Genome sequences of 191 LAB and 225 Vibrionaceae strains with complete genome level as indicated in NCBI database website, including all *Lactip. plantarum*, *Lactococcus lactis*, *Lacto. garvieae*, *Weissella hellenica*, *Pediococcus pentosaceus*, *Leuconostoc pseudomesenteroides*, *V. parahaemolyticus*, *V. harveyi*, *V. campbellii*, *V. rotiferianus*, *V. alginolyticus*, *V. cholerae*, *V. anguillarum*, *V. vulnificus* and *Photobacterium damselae*, were downloaded from RefSeq database^18^ and used as reference sequences. After orthogroup inference, functional gene annotation, and preclusion of those related to regular basic biological processes, the sole remaining shared gene orthogroup possessed by both *Lactiplantibacillus*^17^ and *Vibrio* is related to quorum sensing. ‘–’ indicates not available. Shared gene orthogroups with protein identity lower than 90% are not shown.

The genome information and genome-based phylogeny of *Lactip. plantarum* strain L75a and *V. parahaemolyticus* strain 64 are displayed in Extended Data Fig. 7.

### Are the probiotic metabolites attributed to the benefit or detriment of the probiotic to the pathogen?

**Figure 3:**
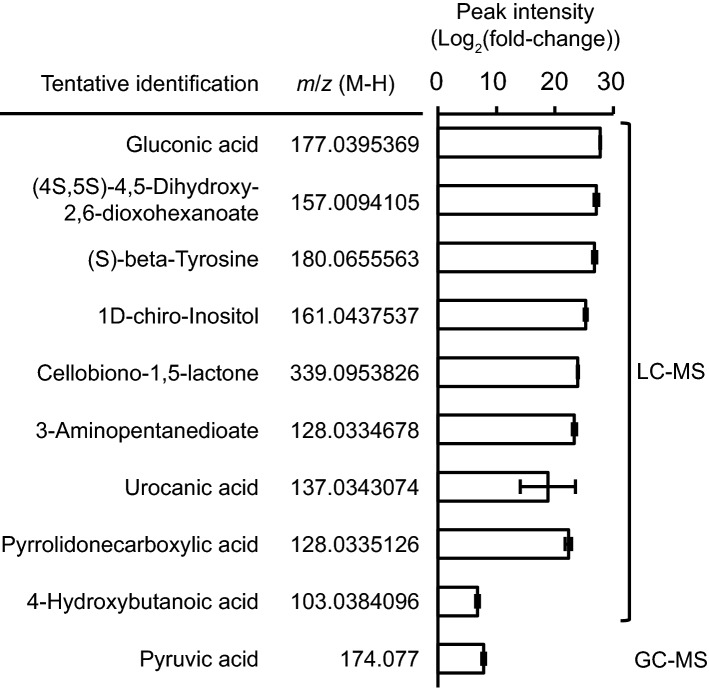
Ten most significantly abundant metabolites detected from the extract of cell-free fermented broth of *Lactip. plantarum* strain L75a. To distinguish compounds affiliated to the medium from the metabolites excreted by *Lactip. plantarum*, a set of MRS broth cultures without inoculation of the isolate was included as ‘control’. The pyruvic acid was detected by GC-MS and the other 9 metabolites by LC-MS. The fold-change was calculated by comparing the peak intensity of each metabolite in the cell-free fermented broth of *Lactip. plantarum* to the mean peak intensity of the corresponding compound in the control. The significance of the abundance of these 10 metabolites was determined by the fold-change of peak intensity (fold-change > 100). Error bars represent S.E.M. of LC-MS (*N* = 5) and GC-MS (*N* = 6) analyses.

Concerning the probiotic metabolites, the liquid chromatography-mass spectrometry (LC-MS) and gas chromatography-mass spectrometry (GC-MS) identified 10 significantly abundant metabolites in the extracellular cell-free fermented broth of *Lactip. plantarum* strain L75a when their peak intensity was compared with the control (fold-change > 100). These 10 metabolites included gluconic acid, (4S,5S)-4,5-dihydroxy-2,6-dioxohexanoate, (S)-beta-tyrosine, 1D-chiro-inositol, cellobiono-1,5-lactone, 3-aminopentanedioate, urocanic acid, pyrrolidonecarboxylic acid, 4-hydroxybutanoic acid, pyruvic acid (Fig. 3).

As the potential inhibitory agents^20–25^, gluconic acid, tyrosine, pyrrolidinone and butyric acid-related compounds might be detrimental to *V. parahaemolyticus* via direct inhibition, and the last compound(s) might also act via inhibiting quorum sensing of the pathogen. Since the concentration of each metabolite was consistent in all corresponding treatments, they could jointly express the same level of antagonism against *V. parahaemolyticus* at the higher and lower densities.

As the potential environmental signals^26–28^, the glutamate-derived compound(s) might be conducive to the virulence of *V. parahaemolyticus* either dependently or independently of quorum sensing mechanism, while for tyrosine-derived compound(s), such mechanism is needed; moreover, the pyruvate-derived compound(s) might be conducive or suppressive to *V. parahaemolyticus* depending on the cell density of the pathogen via quorum sensing pathway.

Further, as *Lactip. plantarum* strain L75a and *V. parahaemolyticus* strain 64 do not exploit the same carbon sources (Extended Data Table 3), they thus exhibit no known competitive relationship with respect to their nutritional niche.

### Are the integrated genetic and metabolic aspects attributed to the benefit or detriment of the probiotic to the pathogen?

With regard to the (co-)evolution of species’ niches of *Lactip. plantarum* strain L75a and *V. parahaemolyticus* strain 64, as they possess overlap in the quorum sensing-related genetic niche (Table 1), the virulence of the pathogen at higher and lower density might be contained or aggravated (Fig. 2a,b) by the probiotic-pathogen inter-genus quorum sensing without transforming the commensal microbiome configuration in the host (Fig. 2c).

i) The probiotic-pathogen inter-genus quorum sensing: pathogen at a higher density
  In the treatment of *V. parahaemolyticus* strain 64 at a higher density accompanying *Lactip. plantarum* strain L75a (Fig. 2), detection of the signal molecules (e.g. autoinducer-2, Table 1) and metabolites (e.g. pyruvate-related compound(s), (Fig. 3)) from *Lactip. plantarum* by the quorum sensing receptors might ‘lead to virulence gene repression’^27,29,30^ in *V. parahaemolyticus*. Meanwhile, *Lactip. plantarum* might be triggered to antagonize *V. parahaemolyticus* only when the pathogen is at a higher density (higher levels of autoinducers from the pathogen) compared to that at a lower density; so, there might be a confrontation/competitive balance between the probiotic and the higher-density pathogen, which circumscribed/surpassesed the viciousness of *V. parahaemolyticus* and had as a result that the zebrafish was healthy (Fig. 2a,b). All these pathways might rely on the regulation by the *luxS* gene, which is located at the overlap in the genomes of *Lactip. plantarum* strain L75a and *V. parahaemolyticus* strain 64 (Table 1).
ii) The probiotic-pathogen inter-genus quorum sensing: pathogen at a lower density
  According to literatures, the signal molecules are capable of inducing virulence of *Vibrio* at a low cell density^29^ and furnishing the inter-genus communication of *Lactiplantibacillus* and *Vibrio* via quorum sensing^15,17^; so, comparing to the treatment of the pathogen at a higher density with the probiotic strain, in the treatment of *V. parahaemolyticus* strain 64 at a lower density accompanying *Lactip. plantarum* strain L75a (Fig. 2), the autoinducer(s) (Table 1) and metabolites (Fig. 3) secreted by *Lactip. plantarum* might ‘activate virulence gene expression,^27,29,30^ in *V. parahaemolyticus*, which jeopardized the health of *D. rerio* (Fig. 2a,b). Interestingly, these virulence regulation in *Vibrio* at molecular, genetic and chemical levels are related to the *luxS* gene regulation (Table 1).

Moreover, since the quorum sensing-regulated luminescence (of the type strain *V. campbellii* BB120, Extended Data Fig. 3a) and virulence^14,17^ of *Vibrio* could be spurred by the cells of *Lactiplantibacillus*, the intimate cell-to-cell contact of *Lactip. plantarum* strain L75a and *V. parahaemolyticus* strain 64 might be another contributor to compromise the host *D. rerio*.

### Concluding remarks

Probiotics might be beneficial to pathogens, but also detrimental. Generally, the efficacies of currently-good ‘probiotics’ are assessed under constricted experimental scenarios, which are deficient in providing extensive facets to discover their long-term and widespread influences. Whether or not the application of a probiotic would mitigate or exacerbate the virulence of pathogens towards versatile hosts, e.g., a sub-healthy/slightly compromised host or a diseased/highly stressed host (as evoked by the pathogen at a lower or higher density, respectively), is still pending for clever investigations on the fitness of the probiotics. Indeed, the beneficial activities of probiotics are tricky to maintain and regulate. To achieve by means of probiotics improvement of human and animal well-being, careful attention to and in-depth reseach of the probiotic-pathogen interaction is warranted.

Meanwhile, to safeguard the use of probiotics, rigorous rules and regulations are obligatory to stipulate the stringent manufacturing and formulation benchmarks^31,32^. To secure ‘life below water’ of the ‘FAO sustainable development goals’, scientists are urging to implement microbiome studies and microbial management on aquatic animals and their living environment^33,34^. Recently, FAO has raised ‘bolster trade and food safety standards’ as one of the seven priority areas to minimise the detrimental effects of COVID-19^35^. Our study is informative for organisations, such as FAO, OIE and WHO, to improve their guidelines to regulate and standardize probiotic assessments, utilization and production.

## Methods

### Media preparation

Except the Vibrionaceae semi-selective TCBS agar (Guangdong Huankai Microbial Sci. & Tech. Co., Ltd., Guangzhou, Guangdong, China) and heat sensitive chemicals, other media and solutions used in this study were prepared in ultrapure water and autoclaved at 121 °C for 15 min before use; agar containing media were supplemented with 1.5–1.8% agar (BioFroxx GmbH, Einhausen, Germany), unless mentioned otherwise. The Luria–Bertani broth/agar, LB0, LB5, LB10 or LB35 broth/agar, consisted of 0.0%, 0.5%, 1% or 3.5% NaCl, respectively; 1% tryptone (Oxoid Ltd., Hampshire, England); 0.5% yeast extract (Oxoid Ltd., Hants, UK); and without/with agar.

### Isolation of lactic acid bacteria (LAB) and Vibrionaceae

*Litopenaeus vannamei* and the rearing water from each of the four farms in Gruangdong Province, China, and *Larimichthys polyactis*, *Odontamblyopus rubicundus*, *Takifugu ocellatus*, *Penaeus monodon*, *Scylla serrata* and water from The South China Sea were collected in more than one replicate. These samples were either handled immediately after collection (within 30 min) or kept in ice and handled within 3–4 h. Small portion of each of the four organs (muscle, intestine, hepatopancreas and gill) from *Li. vannamei* and the intestine from the other aquatic animals were excised and put into a 1.5-ml sterile tube containing 100 μl 0.8% saline and three glass beads of Ø 3 mm. Each organ was homogenized by a Biologix® vortex mixer (Jinan, Shandong, China) for 1–5 min at maximal speed.

To isolate LAB, 5 μl or 1–2 μl of each homogenized organ was spread-plated (in triplicate) or spot-inoculated (in 12 replicates), respectively, on the LAB semi-selective medium, MRS broth (Guangdong Huankai Microbial Sci. & Tech. Co., Ltd., Guangzhou, Guangdong, China) supplemented with agar (MRS agar); the water samples were inoculated directly on MRS agar with the same methods. *Li. vannamei* and the rearing water of Farm 1 was not dedicated for LAB isolation.

To isolate Vibrionaceae, 5 μl of each homogenized organ was spread-plated on TCBS agar in triplicate, or, the bacteria were collected by spearing a sterile needle in each of the organs of *Li. vannamei* and gently streak-plating on TCBS agar in one replicate; the water samples were inoculated directly on TCBS agar with the same methods, or a water droplet of 2–30 μl was spread-plated on TCBS agar.

The MRS and TCBS agar plates were incubated at 27 ± 3 °C for 24–48 and 18 h, respectively. From each colony morphology (colour and shape) of each water sample or of each organ of each aquatic animal, 2–5 (or one, if not sufficient) single colonies were picked from the MRS and TCBS agar, and purified by streak-plating on MRS and 1/10^th^ strength LB5 / LB10 agar, respectively. Each purified isolate was grown in MRS and LB10 broth, respectively, and preserved in 20–40% glycerol (Sigma-Aldrich, Co., MO, USA) at − 80 °C.

### Identification and 16S rRNA gene-based phylogeny of LAB and Vibrionaceae isolates

Firstly, after preservation in 20–40% glycerol at − 80 °C, isolates from MRS agar that were capable of reviving in MRS and LB0 broth, and isolates from TCBS agar in LB5 and LB10 broth were sent to BGI (Shenzhen, Guangdong, China) for 16S rRNA gene sequencing by using only the forward primer to initiate the synthesis of the fragments from the 3′ end. After the fragments were analysed by BLASTn^36^ on the National Center for Biotechnology Information (NCBI) database website (www.ncbi.nlm.nih.gov/), all isolates identified as LAB or Vibrionaceae (Extended Data Table 1) were sent for 16S rRNA gene sequencing by using the reverse primer to initiate the synthesis of the fragments from the 5′ end. For each isolate, the two fragments from the 3′ and 5′ ends with good quality were aligned to generate a contig. As reference sequences, 16S rRNA gene sequences with good quality of 268 LAB type strains, including all 205 '*Lactobacillus*' (this term represents the former genus name which has been reclassified into 25 genera as proposed by Zheng and colleagues^17^), 19 *Leuconostoc*, 13 *Lactococcus*, 12 *Pediococcus* and 19 *Weissella* strains, and 139 Vibrionaceae type strains, including all 103 *Vibrio*, 6 *Aliivibrio*, 4 *Enterovibrio* and 26 *Photobacterium* strains, were obtained from the Ribosomal Database Project^37^ (RDP, http://rdp.cme.msu.edu/). All the 16S rRNA gene sequences (≥ 1200 bp) of the isolates and type strains were subjected to phylogenetic analyses with ClustalW in MEGA7^38^ to generate the neighbour-joining^39^ consensus trees for LAB and Vibrionaceae, with 1000 bootstrap replicates^40^. The evolutionary distances were calculated by the Kimura-2-parameter model^41^ and the non-uniformity of evolutionary rates amongst sites were determined using discrete Gamma distributions.

### Motility and enzymatic characterization of 28 LAB and 37 Vibrionaceae isolates

According to the 16S rRNA gene-based phylogenetic analyses, 28 LAB and 37 Vibrionaceae isolates, which represented proportionally to the abundance, diversity and origin (including location, host, environment and organ) among all LAB and Vibrionaceae isolates, respectively, were selected for functional characterization. These isolates were inoculated in MRS and LB10 broth, respectively, and incubated at 200 r.p.m. at 28 °C for 1–3 days. The cells of each culture were washed once by centrifugation at 5000 × *g* for 5 min at room temperature to remove the supernatant and the cell pellets were resuspended in ultrapure water (for LAB isolates) and 1% saline (for Vibrionaceae isolates). After determination of the cell density of each suspension with a spectrophotometer (BioTek® Synergy™ HTX multi-mode reader, BioTek Instruments, Inc., Winooski, VT, USA) at 600 nm (OD_600_), each cell suspension was adjusted to a final density of 10^9^ cells ml^−1^, and 3 μl of each suspension was spot-inoculated on each medium in triplicate and each assay was performed aerobically at 28 °C. The adjusted protocols will be mentioned otherwise.

The swimming and swarming media were prepared in MRS (for LAB isolates) and LB10 (for Vibrionaceae isolates) broth supplemented with 0.3% (w/v) and 0.6% (w/v) agar, respectively. The control medium for Vibrionaceae was LB10 broth supplemented with 1.5% (w/v) agar. 2 μl of each cell suspension was spot-inoculated at the centre of each medium. The swimming and swarming plates for LAB and Vibrionaceae isolates were incubated at 26 ± 2 °C for 42 and 18 h, respectively. After incubation, the swimming and swarming activities were determined by measurement of the motility outgrowth from the inoculum point.

The amylase indicator media for LAB and Vibrionaceae isolates consisted of 1% (w/v) wheat powder and LB0 agar supplemented without and with 1% NaCl, respectively. The agar plates were incubated for 14 and 2 days, respectively.

The lipase indicator media for LAB and Vibrionaceae isolates consisted of MRS and LB10 agar supplemented with 0.6% lecithin from soybean, respectively. The agar plates were incubated for 7 days and 2 days, respectively.

The protease indicator media for LAB and Vibrionaceae isolates consisted of 1.5% Difco™ skim milk powder (BD, Franklin Lakes, NJ, USA) and agar supplemented without and with LB10 broth, respectively. The agar plates were incubated for 7 days and 1 day, respectively.

The indicator media of feed degradation consisted of 1% (w/v) ground and sieved (through a sieve with the pore size of 425 μm) shrimp feed (type: No. 0, Guangdong Yuehai feed group, Guangdong, China) supplemented without and with 1% NaCl for LAB and Vibrionaceae isolates, respectively. The agar plates were incubated for 7–8 days.

The faeces of *Li. vannamei* was firstly sun-dried and then dried in an oven at 60 °C for 24 h. The shell and muscle of *Li. vannamei* were dried in an oven at 105 °C for 6 h. After drying, each matter was ground, sieved (through a sieve with the pore size of 425 μm), weighed, put in glass bottles and autoclaved. Afterwards, each sterile matter was dried at 60 °C until it was visibly dried and the condensation inside the bottle disappeared. Then, each matter was proportionally mixed with the autoclaved 1.5% agar (w/v) supplemented without and with 1% NaCl, to test the corresponding degradation activity of the LAB and Vibrionaceae isolates, respectively. Each matter was suspended at 1% (w/v) in each medium. For LAB and Vibrionaceae isolates, the faeces agar plates were incubated for 14 and 7 days, respectively; the shell and muscle agar plates for 14 and 2 days, respectively.

The corresponding enzymatic activity was determined by measurement of the degradation halo around and/or within the inoculum. Vague or weak activity was considered as positive for the corresponding activity.

### Production of LAB-fermented both

To produce LAB-fermented both, a pre-grown culture of each LAB isolate was inoculated into fresh MRS broth to reach an initial density of 10^5^ cells ml^−1^. The liquid portion of each culture took 10% of the volume inside the container and each culture was incubated at 200 r.p.m. at 28 °C for 24 h. The final density of each fermented broth was approximately 10^9^ cells ml^−1^. As a control treatment, the MRS broth was not inoculated with LAB.

To collect the cell-free fermented broth of each isolate, each whole fermented broth was centrifuged, and the supernatant was filter-sterilized through a 0.22-µm Millex® syringe-driven filter unit (Merck Millipore Ltd., Co. Cork, Ireland) and then stored at − 20 °C before use.

### Antagonism of the metabolites of the 28 LAB isolates against the 37 Vibrionaceae isolates and the type strain *V. campbellii* BB120

The antagonism indicator assay aimed at testing the impact of the cell-free fermented broth of each of the 28 LAB isolates on the proliferation of each of the 37 Vibrionaceae isolates and on the type strain *V. campbellii* BB120 (ATCC® BAA-1116). To imitate the dosage range of the already-applied/commercial LAB fermented broth under the realistic farming conditions, each cell-free fermented broth and the non-inoculated MRS broth (control treatment) was diluted 10^4^-, 10^5^- and 10^6^- times with LB10 broth. The aforementioned cell suspension (10^9^ cells ml^−1^) of each Vibrionaceae isolate was added to each diluted cell-free fermented broth to make a culture mixture with an initial density of 10^5^ cells ml^−1^. 100 μl of each culture mixture was added into a well of a Costar® 96-well cell culture plate (Corning Incorporated, Corning, NY, USA). Each treatment was performed in triplicate. The standing cultures were incubated at 28 °C and OD_600_ values were determined after incubation for 0, 6, 9, 12, 15 and 24 h. The proliferation of a Vibrionaceae isolate treated with a diluted cell-free fermented broth was determined as inhibited or stimulated when the mean OD_600_ value of such culture was consistently at least 0.1 lower or 0.2 higher than that of the corresponding control culture during the exponential growth phase within 24 h, respectively. A similar experimental setup was exploited to test the impact of the metabolites on the proliferation of the type strain *V. campbellii* BB120: each cell-free fermented broth and the non-inoculated MRS broth (control treatment) were diluted with LB35 broth accordingly, and the OD_600_ values were measured after incubation for 0, 4, 6, 8, 10, 12, 16, 20 and 24 h.

### Impact of the fermented broths of the three representative LAB isolates on the bioluminescence and proliferation of *V. campbellii* BB120

Three representative LAB isolates, *W. hellenica* strain L43, *Lactip. plantarum* strain L75a and *Lacto. lactis* strain L80, were selected for further investigation.

The bioluminescence indicator assay was conducted to test the impact of the cell-free supernatant, the cells and the whole fermented broth of each representative isolate on the bioluminescence of the type strain *V. campbellii* BB120. The cells of *V. campbellii* BB120 were washed once and resuspended in 1% saline to a final density of 10^9^ cells ml^−1^. The cell-free and whole fermented broths were prepared as described above and the control treatment was the non-inoculated MRS broth. The cells of each fermented broth were collected by centrifugation and resuspended in ultrapure water of the same volume, and the control treatment was 1% saline. All the tested cell-free supernatants, the cells, the whole fermented broths and the MRS broth were not luminescent; each of them was diluted 50- and 100-times with 1% saline. The cell suspension of *V. campbellii* BB120 was added to each diluted content to make a culture mixture with an initial density of 10^8^ cells ml^−1^ of *V. campbellii* BB120. 100 μl of each culture mixture was added into a well of a 96-well white cell culture plate (Thermo Scientific Nunc, Thermo Fisher Scientific, Rochester, NY, USA). Each treatment was performed in triplicate. Each culture was measured within 15 min after the culture mixture was prepared. The gain value was set at 135.

To verify whether the decrease in bioluminescence was due to growth inhibition of *V. campbellii* BB120, after the bioluminescence was determined, the growth (OD_600_) of *V. campbellii* BB120 was measured after 24 h of incubation.

### Safety evaluation of the three representative LAB isolates (I)

The cells of each representative LAB isolate, *W. hellenica* strain L43, *Lactip. plantarum* strain L75a and *Lacto. lactis* strain L80, were pre-grown, washed once, resuspended in ultrapure water and adjusted to a density of 10^9^ cells ml^−1^. Each cell suspension was used in the following tests for safety evaluation. Each treatment was performed aerobically in triplicate and incubated at 28 °C. The adjusted methods will be mentioned otherwise.

To test the haemolytic activity, the cells of each isolate were washed three times and each cell suspension was diluted with ultrapure water to a density of 10^4^ cells ml^−1^. 5 μl of this diluted cell suspension was spot-inoculated on Columbia CNA blood agar (Guangdong Huankai Microbial Sci. & Tech. Co., Ltd., Guangzhou, Guangdong, China). The cultures were incubated anaerobically at 37 °C for 24 h in zipper-seal pouches (Mitsubishi Gas Chemical Company, Inc., Tokyo, Japan) with AnaeroPack®-Anaero O_2_-absorbing and CO_2_-generating agent (Mitsubishi Gas Chemical Company, Inc., Tokyo, Japan). The α, β or γ haemolytic activity was determined afterwards.

The biofilm formation assay was conducted in 50-ml glass tubes (Ø2.5 cm) containing 20 ml MRS broth each. Each cell suspension was added to the medium to reach an initial density of 10^7^ cells ml^−1^. After the standing cultures were incubated for 14 days, the biofilm formation activity was determined by observation of cell attachment to the solid surface of the tube.

The gelatinase indicator media consisted of LB0 broth supplemented with 12% and 15% gelatine (w/v, Gelatine medium (Guangdong Huankai Microbial Sci. & Tech. Co., Ltd., Guangzhou, Guangdong, China)), respectively, or without gelatine (control treatment). Each medium was filled into a 14-ml Falcon® round-bottom tube (Ø1.5 cm, Corning Incorporated, Corning, NY, USA) and each cell suspension was added to each medium to reach an initial density of 10^7^ cells ml^−1^. Each treatment was performed in triplicate in two sets and incubated at 200 r.p.m. After incubation for respectively 24 and 48 h at 28 °C, one set was statically incubated at 4 °C for 30 min and then the gelatinase activity was determined by observation of liquefied gelatine.

The ammonia-production indicator medium was prepared by adding 40% sterile urea solution (Guangdong Huankai Microbial Sci. & Tech. Co., Ltd., Guangzhou, Guangdong, China) to the autoclaved urea agar base (Guangdong Huankai Microbial Sci. & Tech. Co., Ltd., Guangzhou, Guangdong, China) to reach the final concentration of 1.9% (v/v). After spot-inoculation of each cell suspension (washed three times), the cultures were incubated at 37 °C for 14 days anaerobically according to the aforementioned methods. The positive and negative activities of ammonia production were reflected by red/pink and yellow colors in the agar, respectively.

To test the mucinase activity of the isolates, the mucin indicator assay was prepared by supplementing 2% mucin from porcine stomach (w/v, NM4825, Hefei Bomei Biotechnology Co., Ltd., Hefei, Anhui, China) or 2% glucose (w/v, positive control, Tianjin Fuchen Chemical Reagents Factory, Tianjin, China) in the adjusted MRS broth (10 g l^−1^ tryptone, 10 g l^−1^ beef extract (Huankai Microbial Sci. & Tech. Co., Ltd., Guangzhou, Guangdong, China), 5 g l^−1^ yeast extract, 5 g l^−1^ Sodium acetate anhydrous, 2 g l^−1^ dipotassium hydrogen phosphate trihydrate, 0.58 g l^−1^ magnesium sulfate heptahydrate, 0.25 g l^−1^ manganese sulfate monohydrate). The media were filter-sterilized through 0.22-μm filters before use. The negative control medium was the adjusted MRS broth. Each cell suspension was added to each medium to make a culture mixture with an initial density of 10^6^ cells ml^−1^. 100 μl of each culture mixture was added into a well of a 96-well cell culture plate. Each treatment was performed in quadruplicate. OD_600_ values of the standing cultures were determined after incubation for 24 and 48 h.

To test the cytotoxicity of the cell-free supernatant and cells of the fermented broth of each isolate on the Epithelioma Papulosum Cyprini (EPC), 100 μl EPC (6 × 10^5^–1 × 10^7^ cells ml^−1^) was added into each well of a 96-well cell culture plate and incubated for 24 h before use. The cell-free fermented broth of each isolate was added into each well to reach the 10-, 10^2^-, 10^3^-, 10^4^-, 10^5^- and 10^6^-times dilutions of the original fermented broth. The cells of each isolate were washed three times and added into each well to the final densities of 10^3^, 10^4^, 10^5^, 10^6^, 10^7^ and 10^8^ cells ml^−1^. After the standing cultures were incubated for 24 h, 50 μl of one-time diluted MTT solution (MTT Cell Proliferation and Cytotoxicity Assay Kit, Jiangsu Keygen Biotech Corp., Ltd., Nanjing, Jiangsu, China) was added into each well and then the cultures were incubated for 4 h. The next procedures were conducted according to manufacturer’s protocol. Finally, the OD_490_ values were determined. The control treatment was the untreated EPC.

To prepare the bile salt and pH tolerance assays, the bile salts (No. 3, Guangdong Huankai Microbial Sci. & Tech. Co., Ltd., Guangzhou, Guangdong, China) were added to LB0 broth to the final concentrations of 0.0%, 0.1%, 0.2%, 0.3%, 0.5%, 1%, 1.5%, 2%, 2.5% and 3% (w/v), respectively, and the LB0 broth was diluted to 70% strength; the pH values of MRS broth was adjusted to 1, 2, 3, 4, 5, 6 and 7 by addition of HCl or NaOH. Each cell suspension was pre-mixed with each indicator medium to make a culture mixture with an initial density of 10^7^ cells ml^−1^. 800 μl of each culture mixture of the bile salt indicator assay was added to a well of a Costar® 24-well cell culture plate (Corning Incorporated, Corning, NY, USA), and 100 μl mixture of the pH indicator assay was added to a well of a 96-well cell culture plate. Each treatment was performed in quadruplicate and sextuplicate, respectively. The OD_600_ values were determined every 1–2 days for a duration of 5 days and every day for 2 days, respectively.

### Safety evaluation of the three representative LAB isolates (II)—testing for antibiotic susceptibility

69 antibiotic discs were used. The cell suspension of *W. hellenica* strain L43 (30 μl), *Lactip. plantarum* strain L75a (10 μl) or *Lacto. lactis* strain L80 (10 μl) was spread-plated on each MRS agar plate (Ø6 cm). The cultures were incubated at 28 °C for 61 ± 1 h before scoring. The test was conducted in triplicate.

### Pre-cultivation, transfer and acclimatization of *Li. vannamei* shrimp

Upon arrival in the aquaculture laboratory, the *Li. vannamei* were pre-cultivated in the rearing water containing 0.5% seawater crystal (Yanzhibao™, Guangzhou, Guangdong, China) in a recirculation aquaculture system (RAS) for at least 14 days before use. The testing system was located in a room at the ambient temperature of 28 ± 2 °C. 10 *Li. vannamei* of approximately 4 cm were gently introduced to each opaque testing unit (Ø18.9 cm) and allowed to acclimatize for at least 4 h. Each testing unit contained 3 L of RAS water and was covered with an opaque lid to maintain darkness and insulation without blocking aeration, which was supplied continuously by an air stone connected to an air pump.

### Safety evaluation of the three representative LAB isolates (III)—pathogenicity to *Li. vannamei*

The fermented broth of *Lactip. plantarum* strain L75a was added to each testing unit to reach 10^3^-, 10^4^-, 10^5^- and 10^6^-times dilution, and that of *W. hellenica* strain L43 and *Lacto. lactis* strain L80 to reach 10^3^- and 10^4^-times dilution. Each treatment was performed in triplicate. To determine the LAB cell density, 5 μl rearing water from each testing unit was spot-inoculated on MRS agar in triplicate every day. Each *Li. vannamei* received on average one feed pellet every day. The survival of *Li. vannamei* was determined after 5 days. No fermented broth was added to the control treatment.

### Screening for the most virulent *Vibrio* isolate in *D. rerio* bioassays

In the first test, *V. parahaemolyticus* strains 62, 64, 220, 228 and 247, *V. cholerae* strain 41, *V. anguillarum* strain 150a, *V. atypicus* strain 180, *V. harveyi* strain 1, *V. vulnificus* strain 73 and *V. shilonii* strain 89, representing those from the six biggest phylogenetic clades of *Vibrio* species (Extended Data Fig. 1b), were tested (Extended Data Fig. 5c). Upon arrival, 10 zebrafish were introduced to each testing unit (holding 3 L of dechlorinated tap water) and allowed to acclimatize for at least 6 h. On day 0, 3 *Li. vannamei* (as the feed and infection stimulus) of 5–6 cm were washed with sterile water, introduced to a 15-ml cell suspension of each *Vibrio* isolate with a density of 10^9^ cells ml^−1^ and incubated for 2 h. Then, each of these co-cultures was added to each corresponding testing unit. Each treatment was performed in one replicate. Dead fish were removed every day and aeration was paused intermittently. The survival of *D. rerio* was scored after cultivation for 7 days.

From this point onwards, the salinity of the rearing water (dechlorinated tap water) of *D. rerio* used in the following bioassays was raised to 5 ppt by gradually adding seawater crystal everyday.

Next, the three most virulent isolates, *V. harveyi* strain 1, *V. parahaemolyticus* strain 64 and *V. anguillarum* strain 150a, were further tested for their virulence at various densities in the rearing water. Every day, the cells of each isolate were washed once and added to the 2-L rearing water to reach the densities of 5 × 10^5^, 2.5 × 10^6^ and 10^7^ cells ml^−1^, respectively; approximately 45 feed pellets on average were added to each testing unit. One *Li. vannamei* (as the feed and infection stimulus) of 6–7 cm was introduced to each testing unit on day 5. Each treatment was performed in one replicate and no isolate was added to the control treatment. Dead fish were removed when observed. Aeration by an air pump was not provided. The survival of *D. rerio* was scored after cultivation for 15 days.

### Impact of *Lactip. plantarum* strain L75a on the proliferation of *V. parahaemolyticus* strain 64 *in vitro*

The cell-free and whole fermented broths of *Lactip. plantarum* strain L75a were prepared as described above; the cells of the fermented broth were collected by centrifugation and resuspending in the same volume of 0.5% saline; each of them was diluted 10^2^-, 10^3^- and 10^4^-times with 0.5% saline. The cells of *V. parahaemolyticus* strain 64 were pre-grown in LB5 broth, washed once, resuspended in 0.5% saline, and added to each of the three diluted contents of the fermented broth to make a culture mixture with an initial *Vibrio* density of 10^5^ cells ml^−1^. In the control treatments, the *Vibrio* cells were treated with 0.5% saline only. Each treatment was performed in triplicate. The standing co-cultures were incubated at 26 ± 2 °C and determined for the colony forming unit (CFU) on TCBS and/or MRS agar in triplicate after incubation for 0, 4 and 8 h.

### Impact of *Lactip. plantarum* strain L75a against *V. parahaemolyticus* strain 64 on *D. rerio*

#### Survival of *D. rerio*

To prepare the feed and infection stimulus, each piece of shrimp muscle (0.5 g approximately) was cleaned with sterile water and placed into a 20-ml cell suspension of *V. parahaemolyticus* strain 64 (10^4^ or 10^6^ cells ml^−1^) for 30 min before use.

On day 0, 2 and 4, the cells of *V. parahaemolyticus* strain 64 were washed once and added to each testing unit, containing 10 *D. rerio* (Tuebingen from Pearl River Fisheries Research Institute, Chinese Academy of Fishery Sciences, Guangzhou, Guangdong, China) in 3.5-L rearing water, to reach the densities of 10^4^ (lower density) and 10^6^ (higher density) cells ml^−1^, respectively; the aforementioned pre-treated shrimp muscle was introduced to the corresponding rearing water, respectively. On day 2 and 4, the water containing lower and higher cell densities of *V. parahaemolyticus* strain 64 was supplemented with or without the 10^4^-times-diluted fermented broth of *Lactip. plantarum* strain L75a. The *D. rerio* in the two control treatments were untreated or treated only with the10^4^-times-diluted fermented broth. Each treatment was conducted in triplicate. pH level was stabilized at 7.8 ± 0.1 during the entire test, as measured by a pH meter (PH-220 W, Hangzhou Qiwei Instrument Co., LTD, Hangzhou, Zhejiang, China). Dead fish were removed when observed. Aeration from an air pump was provided continuously during the entire test. Scoring was conducted after 9 days of cultivation when the fish survival of at least two replicates of the same treatment was just down to 50%.

### *Bacterial community in D. rerio*

Immediately after the fish survival was scored, four fish of each testing unit were collected and anesthetized by ice water; the muscle, liver and intestine of every two fish were excised and mixed in one RNAase-free tube, respectively. Therefore, the fish of each treatment were sampled in sextuplicate. The samples were stored at − 80 °C until further processing. DNA extraction, PCR amplification of the 16S rRNA gene (with an inclusion of 0.2 µl BSA within each 20-µl mixture for the PCR reaction), Illumina MiSeq sequencing and processing of the sequencing data were performed by Majorbio Bio-Pharm Technology Co. Ltd. (Shanghai, China) according to the protocols described by Wang and colleagues.^42^

### *Histology of D. rerio*

A similar *D. rerio* bioassay was conducted in a smaller scale (one replicate per treatment) to collect the fish tissues. On the day when the survival of anyone of the treatments went just down to 50%, one fish of each treatment was collected and the muscle, liver and intestine were dissected.

To prepare the haematoxylin and eosin (H&E)-stained tissue sections, each organ sample was fixed in Bouin's solution for 3 days at room temperature. Further processing and photomicrography of the sections were conducted by Wuhan Servicebio Technology Co., Ltd (Wuhan, Hubei, China) based on Servicebio’s in-house protocols.

To prepare the tissue sections for transmission electron microscopy (TEM), each organ sample was fixed in 2.5% glutaraldehyde in phosphate buffer (pH7.0) for 10 days at 5 °C. Afterwards, each fixed tissue was post-fixed in 1% osmium tetroxide at room temperature for 3 h and dehydrated with gradient ethanol. Then, each sample was transferred in acetone and embedded in Spurr’s resin (SPI Chem™ Low Viscosity “Spurr” Kits, Structure Probe, Inc., West Chester, PA, USA). A 70-nm ultra-thin section of each sample was cut by a Reichert microtome (Leica em uc7, Wetzlar, Germany) and stained with uranyl acetate and lead citrate. The photomicrographic images of each stained section was captured with a Talos L120C transmission electron microscope (FEI™, Thermo Fisher Scientific, Hillsboro, Oregon, USA) connected to an image analyzer system.

### Genomic DNA extraction and genome sequencing

*Lactip. plantarum* strain L75a and *V. parahaemolyticus* strain 64 were respectively grown in LB0 and LB10 broth at 28 °C for 24 h. The cells of each isolate were washed three times with ultrapure water and the cell pellets were then stored at − 80 °C before further processing. The genomic DNA of each isolate was extracted with the DNeasy® Plant Mini Kit (QIAGEN, Hilden, Germany) according to manufacturer’s instructions. Library preparation and genome sequencing were conducted in Annoroad Gene Technology (Beijing, China) according to the protocols described by Ming and colleagues^43^.

### Comparative genomic analyses

#### Genome assembly and annotation

For the genome sequences of *Lactip. plantarum* strain L75a and *V. parahaemolyticus* strain 64, the SMRT Link v8.0^44^ was used to discard low-quality reads and to control the quality of raw sequence reads to obtain clean sequence reads, which were *de novo*-assembled by MECAT2^45^. Genome annotation was conducted by Prokka^46^.

#### Analyses of orthogroups

Genome sequences of 191 LAB and 225 Vibrionaceae strains with complete level (as shown on NCBI database website) were downloaded from RefSeq database^18^. These 416 reference strains included: all *Lactip. plantarum*, *Lacto. lactis*, *Lacto. garvieae*, *W. hellenica*, *Ped. pentosaceus*, *Le. pseudomesenteroides*, *V. parahaemolyticus*, *V. harveyi*, *V. campbellii*, *V. rotiferianus*, *V. alginolyticus*, *V. cholerae*, *V. anguillarum*, *V. vulnificus* and *Ph. damselae*. Orthogroup inference for the genomes of *Lactip. plantarum* strain L75a, *V. parahaemolyticus* strain 64 and the 416 reference strains was conducted by OrthoFinder^47^.

#### Functional annotation

Functional annotation of genes in the resulted orthogroups was conducted by searching for homology of protein sequences in UniProt (http://www.uniprot.org)^48^ using BLASTp (-max_target_seqs 1 -evalue 1e-5)^49^. The UniProt IDs of each orthogroup were used to link information including protein name, gene name, organism, gene ontology, function and protein family, etc., by using the Retrieve/ID mapping tool (https://www.uniprot.org/uploadlists/)^50^.

#### Analyses of the probiotic-pathogen shared gene orthogroups that might rationalize the benefit or detriment of the probiotic to the pathogen

Next to the aforementioned functional annotation, the shared gene orthogroups of single- and multiple-copy were analysed among *Lactip. plantarum* strain L75a, *V. parahaemolyticus* strain 64 and the 416 reference strains, and also among *Lactip. plantarum* strain L75a and *V. parahaemolyticus* strain 64. Then, the shared gene orthogroups related to regular basic biological processes were excluded and the remaining shared gene orthogroups possessed by both *Lactiplantibacillus* and *Vibrio* species were further analysed for their functions based on literatures.

#### Whole genome-based phylogeny of *Lactip. plantarum* strain L75a and *V*. *parahaemolyticus* strain 64

The resulted orthogroups of *Lactip. plantarum* strain L75a, *V. parahaemolyticus* strain 64 and the 416 reference strains were used to construct a phylogenetic tree with STAG in OrthoFinder^47^, which was then visualized with iTOL (https://itol.embl.de/)^51^. For both tools, default settings were applied.

### Metabolomics of *Lactip. plantarum* strain L75a

The cell-free fermented broth of *Lactip. plantarum* strain L75a was produced in accordance to the aforementioned method and stored at − 80 °C before further processing. MRS culture without *Lactip. plantarum* strain L75a inoculation was included as a control for determination of the background. Metabolite extraction, liquid chromatography mass spectrometry (LC-MS) analyses and gas chromatography mass spectrometry (GC-MS) analyses were conducted by Bionovogene (Suzhou, Jiangsu, China) according to Bionovogene’s in-house protocols, which are described as follows.

#### LC-MS analyses

To extract the metabolites^52–54^, each sample was thawed at 4 °C, vortexed for 30 s and let stand for 2 h. 1.5 ml of each sample was transferred into a new tube, dried with a vacuum concentrator (concentrator plus/vacufuge® plus, 5305, Eppendorf AG, Hamburg, Germany), dissolved in 500 µl methanol (− 20 °C), vortexed for 1 min and centrifuged at 13,780 × *g* at 4 °C for 10 min. After centrifugation, 100 µl supernatant of each sample was transferred to a new tube, supplemented with 400 µl methanol (− 20 °C), vortexed for 1 min and dried with a vacuum concentrator. The dried compounds of each sample were redissolved in 150 µl 2-Chloro-L-phenylalanine (4 ppm)-methanol (80%), and the supernatant was filtered through a PTFE membrane (0.22 µm, Jinteng, Tianjin, China). 20 µl of each filtered sample was pooled and used for quality control and calibration, and the remaining portion was used for LC-MS analyses.

A typical run was constructed based on that described by Want and colleagues^55^. For chromatographic separation, the Vanquish (Thermo Fisher Scientific Inc., Waltham, MA, USA) instrument was equipped with an ACQUITY UPLC HSS T3 column (150 × 2.1 mm, 1.8 μm, Waters Corporation, Milford, MA, USA), which was maintained at 40 °C. The temperature of the autosampler was dedicated at 8 ℃. The mobile phase was comprised of a binary eluent solvent system; for positive ionization mode, the solvents were double distilled water (solvent B2) and acetonitrile (solvent A2), both consisted of 0.1% formic acid (v/v); for negative ionization mode, the solvents were 5 mM ammonium formate (in double distilled water) (solvent B1) and acetonitrile (solvent A1). 2 μl of each sample was injected after equilibration and analysed at a flow rate of 0.25 ml/min in a gradient of solvent A as follows: 2% A2/A1 in 1 min; 2–50% A2/A1 in 8 min; 50–98% A2/A1 in 3 min; 98% A2/A1 in 1.5 min; 98–2% A2/A1 in 30 s; 2% A2 in 6 min (positive mode), 2% A1 in 3 min (negative mode).

The electrospray ionization multistage tandem mass spectrometry (ESI-MS^n^) was implemented on the Q Exactive Focus mass spectrometer (Thermo Fisher Scientific Inc., Waltham, MA, USA) using the following conditions: spray voltage of 3.50 kV and 2.50 kV in positive and negative ion mode, respectively; sheath gas and auxiliary gas at 30 and 10, respectively (arbitrary units); capillary temperature at 325 °C; full-scan resolution 70,000; scan range *m*/*z* 81–1000; acquiring MS/MS data by data-dependent acquisition (DDA) mode; normalized collision energy for higher-energy collisional dissociation (HCD) was 30 eV; the unnecessary information in MS/MS spectra was removed by dynamic exclusion.

Six samples of each treatment (the *Lactip. plantarum* strain L75a-fermented broth and the non-inoculated MRS broth) were used for LC-MS diagnosis. According to the criteria for quality control and quality assurance^52,56^, the pre-processed data were inspected and one fermented broth sample was excluded. Therefore, five replicates were included for the LC-MS analyses.

#### GC-MS analyses

To extract the metabolites^52,54^, each sample was lyophilized, redissolved in 500 µl methanol (− 20 °C), transferred into a new tube and vortexed for 1 min. 60 μl isotope-labelled alanine (10 mM) and 60 μl nonadecanoic acid (0.2 mg ml^−1^) were added as the internal standard. After being vortexed for 1 min and centrifuged at 13,780 × *g* at 4 °C for 10 min, 500–600 µl supernatant of each sample were transferred to a new tube and dried with a vacuum concentrator. After 60 μl *O*-methylhydroxylamine hydrochloride was added to the dried compounds, the mixture was vortexed for 30 s and incubated at 37 °C for 2 h. Then, 60 μl BSTFA (*N*,*O*-Bis(trimethylsilyl)trifluoroacetamide) with 1% TMCS (trimethylchlorosilane) was added. After incubation at 37 °C for 90 min and centrifugation at 13,780 × *g* for 5 min, 90–100 μl supernatant of each sample was collected. 20 µl of each sample was pooled and used for quality control and calibration, and the remaining portion was used for GC-MS analyses.

A typical run was constructed based on that described by Want and colleagues^56^. The Agilent 7890A gas chromatography (Agilent Technologies, Inc., Santa Clara, CA, USA) instrument was equipped with a J&W HP-5MS GC column (30 m × 0.25 mm, 0.25 μm, part number 19091S-433, Agilent Technologies, Inc., Santa Clara, CA, USA) supplied with helium carrier gas at a constant flow rate of 1 ml min^−1^. The following parameters were applied: 1 μl sample was injected by the autosampler in split mode, split ratio 20:1, injection temperature 280 °C, interface temperature 150 °C, ion source temperature 230 °C, full-scan method with scan range *m*/*z* 35–750; the following temperature programs were applied: initial temperature at 60 °C for 2 min, ramp rate at 10 °C min^−1^ to 300 °C, final temperature at 300 °C for 5 min^52,54^.

Six samples of each treatment (the *Lactip. plantarum* strain L75a-fermented broth and the non-inoculated MRS broth) were used for GC-MS analysis and all data fulfilled the criteria for quality control^52^. Therefore, six replicates were included for the GC-MS analyses.

### Exploitation of carbon sources by the *Lactip. plantarum* strain L75a and *V*. *parahaemolyticus* strain 64

The cells of *Lactip. plantarum* strain L75a and *V. parahaemolyticus* strain 64 were washed three times and resuspended respectively in ultrapure water and 1% saline to obtain the density of 10^6^ cells ml^−1^. 130 µl cell suspension of each isolate was added into each well of a EcoPlate™ (Biolog, Hayward, CA, USA). The OD_590_ values were determined after incubation at 28 °C for 5 days.

### Statistics and reproducibility

The impacts of *Lactip. plantarum* strain L75a against *V. parahaemolyticus* strain 64 on the survival and bacterial abundance of *D. rerio* (Fig. 1), of the fermented broths of *W. hellenica* strain L43, *Lactip. plantarum* strain L75a and *Lacto. lactis* strain L80 on bioluminescence of *V. campbellii* BB120 (Extended Data Fig. 3a), of the cell-free supernatant, the cells and the whole fermented broth of *Lactip. plantarum* strain L75a on the proliferation of *V. parahaemolyticus* strain 64 (Extended Data Fig. 6) were compared with one-way analysis of variance and with post hoc Duncan analysis (*P* < 0.05). In Extended Data Fig. 3b, the differences between the proliferation of *V. campbellii* BB120 treated by a fermented broth versus by the corresponding control treatment were analysed by independent-samples *t*-test (*P* < 0.05). In Extended Data Fig. 5a, the pathogenicity of the fermented broths of *W. hellenica* strain L43, *Lactip. plantarum* strain L75a and *Lacto. lactis* strain L80 to *Li. vannamei* were analysed with one-way analysis of variance and with post hoc Duncan analysis (*P* > 0.05). All the statistical tests were two-tailed.

### Ethics statement

All experiments on zebrafish were approved by the Ethics Committee of the Laboratory Animal Center of South China Agricultural University (2019F001). All experiments were performed in accordance with relevant guidelines and regulations. The studies on zebrafish are reported in accordance with ARRIVE (Animals in Research: Reporting *In Vivo* Experiments) guidelines^57^.

## Supplementary Information

Supplementary Information.

## Data Availability

Good-quality sequences of the isolates in this study have been deposited in GenBank. The accession numbers for the 16S rRNA gene sequences of the LAB isolates L1-L82 are MW785576-MW785631, the Vibrionaceae isolates 1–53 are MT974072-MT974089, and the Vibrionaceae isolates 57–259 are MW785632-MW785741, respectively. The accession numbers of the genomes of *Lactip. plantarum* strain L75a and* V. parahaemolyticus* strain 64 are CP074419-CP074423 (BioProject PRJNA716106, BioSample SAMN18394780) and CP074415-CP074418 (BioProject PRJNA716109, BioSample SAMN18394829), respectively.
