# Supplementary material for: Probiotics: their action against pathogens can be turned around
Source: Sci Rep. 2021 Jun 24;11:13247. doi: 10.1038/s41598-021-91542-3 (PMC8225825; doi:10.1038/s41598-021-91542-3)
Supplement: Supplementary file 1 — Supplementary Information. [file 41598_2021_91542_MOESM1_ESM.pdf]

Probiotics: their action against pathogens can be turned around
Supplementary information

Rationale of using *Lactiplantibacillus plantarum* strain L75a and *Vibrio parahaemolyticus* strain 64 to elaborate the positive and negative impacts of the probiotic on the pathogen

To isolate lactic acid bacteria (LAB) and Vibrionaceae, samples of multiple replicates from farmed and wild aquatic animals (including *Litopenaeus vannamei*, *Larimichthys polyactis*, *Odontamblyopus rubicundus*, *Takifugu ocellatus*, *Penaeus monodon*, *Scylla serrata*) and their rearing water and natural water (Extended Data Table 1) were inoculated on LAB and Vibrionaceae semi-selective media, i.e. de Man, Rogosa and Sharpe (MRS) agar and Thiosulfate-Citrate-Bile salts-Sucrose (TCBS) agar, respectively. 56 and 201 isolates were selected from the MRS and TCBS agar, respectively.

According to the BLASTn (Extended Data Table 1) and taxonomic (Extended Data Fig. 1) analyses, a total of 56 and 128 isolates were identified as LAB and Vibrionaceae, respectively. From these isolates, 28 LAB (from the *Weissella*, *Lactococcus*, '*Lactobacillus*' (this term represents the former genus name which has been reclassified into 25 genera as proposed by Zheng and colleagues[1]), *Pediococcus* and *Leuconostoc* clades) and 37 Vibrionaceae (from the *Vibrio* and *Photobacterium* clades) isolates were selected for motility and enzymatic characterization (Extended Data Fig. 2a and 2b); further, the cell-free fermented broth of each of the 28 LAB isolates was diagnosed for the suppressive or conducive impact on the propagation of each of the 37 Vibrionaceae isolates (Extended Data Fig. 2c).

Based on the phylogenetic analyses (Extended Data Fig. 1a) and *in vitro* bioassays (Extended Data Fig. 2), three representative LAB isolates, *Weissella hellenica* strain L43, *Lactip. plantarum* strain L75a and *Lactococcus lactis* strain L80 were selected for further investigation because of the following reasons: i) they represented the three most abundant genera amongst all LAB isolates collected in this study (Extended Data Fig. 1a), ii) they exhibited relatively strong protease activity (Extended Data Fig. 2a and 2b), iii) their diluted cell-free fermented broths were inhibitory to the proliferation of at least four tested Vibrionaceae isolates (Extended Data Fig. 2c), and iv) they grew well on MRS agar and in MRS broth.

To screen for a promising probiotic isolate, several tests were conducted. They revealed that i) the fermented broth of *Lactip. plantarum* strain L75a significantly suppressed the quorum sensing-regulated bioluminescence of the type strain *V. campbellii* BB120 (ATCC® BAA-1116, Extended Data Fig. 3a) without inhibiting its proliferation (Extended Data Fig. 3b); ii) the safety evaluation based on the guidelines published by FAO and WHO[2] demonstrated that *Lactip. plantarum* strain L75a satisfied the basic criteria as a probiotic (Extended Data Fig. 4, Extended Data Table 2); iii) *Lactip. plantarum* strain L75a showed no virulence to *Li. vannamei* (Extended Data Fig. 5a) and persisted longer in the rearing water than *W. hellenica* strain L43 and *Lacto. lactis* strain L80 (Extended Data Fig. 5b). Therefore, *Lactip. plantarum* strain L75a, which is closely related to type strains of terrestrial origin (Extended Data Fig. 1a), was used as candidate probiotic in further in-depth studies.

To screen for the most virulent pathogen, several Vibrionaceae isolates, representing the largest phylogenetic clades of *Vibrio* and *Photobacterium* species (Extended Data Fig. 1b), were selected for pathogenicity tests on *Danio rerio* (Extended Data Fig. 5c) and/or *Li. vannamei* (data not shown). *V. parahaemolyticus* strain 64, which was most virulent to *D. rerio* (Extended Data Fig. 5c), was chosen as

the pathogen in further in-depth studies.

Extended Data

Extended Data Table 1 Information and preliminary classification of the lactic acid bacteria (LAB) and Vibrionaceae isolates. To isolate LAB and Vibrionaceae, the muscle, liver, intestine and gill of aquatic animals, and the rearing and natural water were sampled in more than one replicate. Each sample (organ or water) was inoculated on LAB semi-selective medium (MRS agar) and Vibrionaceae semi-selective medium (TCBS agar), respectively. As selection criteria, 2-5 (or one, if not sufficient) single colonies of each morphology (colour and shape) of each sample were respectively picked from the MRS and TCBS agar and purified. After preservation in 20-40% glycerol at -80 °C, all isolates from MRS agar capable of propagating in MRS and LB0 broth, and all isolates from TCBS agar in LB5 and LB10 broth, were sent for 16S rRNA gene sequencing by using only the forward primer to initiate the synthesis of the sequences from the 3' end. The resulted sequences were analysed by BLASTn[3] on the National Center for Biotechnology Information (NCBI) database website (www.ncbi.nlm.nih.gov/).

Isolate number	Location	Pond/tank* no.	Host/environment	Organ	Query length	Preliminary identification
L1	Farm 3	Pond 1	Water	-	674	LAB
L2	Farm 3	Pond 1	*Litopenaeus vannamei* 1	Hepatopancreas	707	LAB
L4	Farm 3	Pond 1	*Li. vannamei* 1	Intestine	650	LAB
L5	Farm 3	Pond 1	*Li. vannamei* 1	Intestine	673	LAB
L7	Farm 3	Pond 2	*Li. vannamei* 2	Intestine	659	LAB
L8	Farm 3	Pond 2	*Li. vannamei* 2	Intestine	662	LAB
L10	Farm 3	Pond 2	*Li. vannamei* 1	Intestine	664	LAB
L12	Farm 3	Pond 2	Water	-	710	LAB
L14	Farm 3	Pond 2	Water	-	539	LAB
L17	Farm 3	Pond 3	*Li. vannamei* 2	Intestine	693	LAB
L19	Farm 3	Pond 3	*Li. vannamei* 1	Intestine	693	LAB
L20	Farm 3	Pond 4	*Li. vannamei* 2	Intestine	699	LAB
L21	Farm 3	Pond 4	*Li. vannamei* 2	Hepatopancreas	667	LAB
L22	Farm 3	Pond 4	*Li. vannamei* 2	Hepatopancreas	713	LAB
L23	Farm 3	Pond 4	*Li. vannamei* 2	Muscle	635	LAB
L24	Farm 3	Pond 4	*Li. vannamei* 1	Hepatopancreas	659	LAB
L25	Farm 3	Pond 4	*Li. vannamei* 1	Intestine	685	LAB
L26	Farm 3	Pond 4	*Li. vannamei* 1	Intestine	666	LAB
L27b	Farm 3	Pond 4	*Li. vannamei* 1	Intestine	654	LAB
L28	Farm 3	Pond 4	*Li. vannamei* 1	Muscle	683	LAB
L29	Farm 3	Pond 4	Water	-	688	LAB
L30	Farm 3	Pond 4	Water	-	674	LAB
L33	Farm 4	Pond 1	*Li. vannamei* 1	Intestine	668	LAB
L34	Farm 4	Pond 1	*Li. vannamei* 1	Intestine	668	LAB
L37a	Farm 4	Pond 1	*Li. vannamei* 1	Intestine	659	LAB
L37b	Farm 4	Pond 1	*Li. vannamei* 1	Intestine	659	LAB
L38	Farm 4	Pond 1	*Li. vannamei* 1	Hepatopancreas	643	LAB
L39a	Farm 4	Pond 1	*Li. vannamei* 1	Hepatopancreas	607	LAB
L40	Farm 4	Pond 1	*Li. vannamei* 1	Hepatopancreas	702	LAB
L43	Farm 4	Pond 1	*Li. vannamei* 1	Gill	659	LAB
L44	Farm 4	Pond 1	*Li. vannamei* 2	Intestine	633	LAB
L48	Farm 4	Pond 1	*Li. vannamei* 2	Hepatopancreas	633	LAB
L51	Farm 4	Pond 1	*Li. vannamei* 2	Gill	661	LAB
L54	Farm 4	Pond 1	Water	-	633	LAB
L55a	Farm 4	Pond 1	Water	-	490	LAB
L55b	Farm 4	Pond 1	Water	-	666	LAB
L58	South China Sea	-	*Larimichthys polyactis* 3	Intestine	664	LAB
L59	South China Sea	-	*Lar. polyactis* 3	Intestine	667	LAB
L60	South China Sea	-	*Odontamblyopus rubicundus* 1	Intestine	665	LAB
L61	South China Sea	-	*O. rubicundus* 1	Intestine	648	LAB
L64	South China Sea	-	*Takifugu ocellatus* 1	Intestine	673	LAB
L65	South China Sea	-	*T. ocellatus* 1	Intestine	667	LAB
L66	South China Sea	-	*T. ocellatus* 2	Intestine	668	LAB
L67	South China Sea	-	*T. ocellatus* 2	Intestine	670	LAB
L68	South China Sea	-	*T. ocellatus* 2	Intestine	637	LAB
L69	South China Sea	-	*T. ocellatus* 2	Intestine	704	LAB
L70	South China Sea	-	*Penaeus monodon* 3	Intestine	713	LAB
L72	South China Sea	-	*Scylla serrata* 1	Intestine	699	LAB
L73	South China Sea	-	*S. serrata* 1	Intestine	671	LAB
L74	South China Sea	-	*S. serrata* 1	Intestine	668	LAB
L75a	South China Sea	-	*S. serrata* 1	Intestine	656	LAB
L75b	South China Sea	-	*S. serrata* 1	Intestine	679	LAB
L76	South China Sea	-	*S. serrata* 2	Intestine	702	LAB
L79	South China Sea	-	*S. serrata* 3	Intestine	667	LAB

L80	South China Sea	-	*S. serrata* 3	Intestine	698	LAB
L82	South China Sea	-	*S. serrata* 3	Intestine	675	LAB
1	Farm 1	Tank 1-5	*Li. vannamei*	Muscle	726	Vibrionaceae
3	Farm 1	Tank 4-4	*Li. vannamei*	Muscle	725	Vibrionaceae
4	Farm 1	Tank 1-5	*Li. vannamei*	Hepatopancreas	788	Vibrionaceae
5	Farm 1	Tank 4-2	*Li. vannamei*	Hepatopancreas	727	Vibrionaceae
6	Farm 1	Tank 4-2	*Li. vannamei*	Hepatopancreas	789	Vibrionaceae
9	Farm 1	Tank 4-4	*Li. vannamei*	Hepatopancreas	714	Bacillaceae
10	Farm 1	Tank 4-4	*Li. vannamei*	Intestine	726	Vibrionaceae
11	Farm 1	Tank 4-4	*Li. vannamei*	Intestine	715	Vibrionaceae
13	Farm 1	Tank 4-4	*Li. vannamei*	Gill	726	Vibrionaceae
14	Farm 1	Tank 1-5	Water	-	711	Aeromonadaceae
15	Farm 1	Tank 1-5	Water	-	823	Shewanellaceae
16	Farm 1	Tank 1-5	*Li. vannamei*	Intestine	784	Shewanellaceae
17	Farm 1	Tank 1-5	*Li. vannamei*	Intestine	786	Vibrionaceae
18	Farm 1	Tank 1-5	*Li. vannamei*	Intestine	779	Shewanellaceae
19	Farm 1	Tank 1-5	*Li. vannamei*	Intestine	819	Shewanellaceae
21	Farm 1	Tank 2-1	*Li. vannamei*	Muscle	786	Vibrionaceae
22	Farm 1	Tank 2-1	*Li. vannamei*	Intestine	780	Vibrionaceae
23	Farm 1	Tank 1-1	*Li. vannamei*	Gill	783	Shewanellaceae
24	Farm 1	Tank 1-1	*Li. vannamei*	Gill	785	Shewanellaceae
27	Farm 1	Tank 4-4	Water	-	775	Shewanellaceae
28	Farm 1	Tank 4-4	Water	-	795	Aeromonadaceae
29	Farm 1	Tank 4-4	Water	-	803	Vibrionaceae
31	Farm 1	Tank 4-4	*Li. vannamei*	Hepatopancreas	782	Shewanellaceae
34	Farm 1	Tank 3-6	Water	-	799	Vibrionaceae
35	Farm 1	Tank 3-6	Water	-	820	Shewanellaceae
37	Farm 1	Tank 3-6	Water	-	781	Shewanellaceae
38	Farm 1	Tank 4-2	Water	-	768	Moraxellaceae
39	Farm 1	Tank 4-2	Water	-	783	Shewanellaceae
41	Farm 1	Tank 1-2	Water	-	780	Vibrionaceae
43	Farm 1	Tank 2-1	*Li. vannamei*	Hepatopancreas	786	Vibrionaceae
45	Farm 1	Tank 1-2	Water	-	785	Vibrionaceae
47	Farm 1	Tank 4-2	*Li. vannamei*	Hepatopancreas	783	Shewanellaceae
48	Farm 1	Tank 4-2	*Li. vannamei*	Hepatopancreas	782	Shewanellaceae
49	Farm 1	Tank 4-2	*Li. vannamei*	Hepatopancreas	781	Shewanellaceae
50	Farm 1	Tank 1-1	*Li. vannamei*	Hepatopancreas	784	Shewanellaceae
51	Farm 1	Tank 1-1	Water	-	701	Vibrionaceae
52	Farm 1	Tank 1-1	Water	-	775	Shewanellaceae
53	Farm 1	Tank 2-1	*Li. vannamei*	Gill	671	Vibrionaceae
54	Farm 1	Tank 3-6	*Li. vannamei*	Muscle	720	Bacillaceae
55	Farm 1	Tank 3-6	*Li. vannamei*	Intestine	711	Shewanellaceae
56	Farm 1	Tank 4-2	Water	-	744	Enterococcaceae
57	Farm 2	Pond 1	Water	-	784	Vibrionaceae
58	Farm 2	Pond 1	Water	-	789	Vibrionaceae
59	Farm 2	Pond 1	*Li. vannamei* 1	Hepatopancreas	726	Vibrionaceae
60	Farm 2	Pond 1	*Li. vannamei* 1	Hepatopancreas	838	Aeromonadaceae
61	Farm 2	Pond 1	*Li. vannamei* 1	Intestine	779	Vibrionaceae
62	Farm 2	Pond 1	*Li. vannamei* 1	Intestine	788	Vibrionaceae
63	Farm 2	Pond 1	*Li. vannamei* 1	Intestine	781	Shewanellaceae
64	Farm 2	Pond 1	*Li. vannamei* 1	Muscle	734	Vibrionaceae
65	Farm 2	Pond 1	*Li. vannamei* 1	Muscle	825	Shewanellaceae
66	Farm 2	Pond 1	*Li. vannamei* 2	Hepatopancreas	788	Vibrionaceae
67	Farm 2	Pond 1	*Li. vannamei* 2	Hepatopancreas	815	Shewanellaceae
68	Farm 2	Pond 1	*Li. vannamei* 2	Hepatopancreas	784	Shewanellaceae
69	Farm 2	Pond 1	*Li. vannamei* 2	Intestine	823	Aeromonadaceae
70	Farm 2	Pond 1	*Li. vannamei* 2	Intestine	733	Vibrionaceae
71	Farm 2	Pond 1	*Li. vannamei* 2	Intestine	782	Shewanellaceae
72	Farm 2	Pond 1	*Li. vannamei* 2	Intestine	621	Shewanellaceae
73	Farm 2	Pond 1	*Li. vannamei* 2	Muscle	820	Vibrionaceae
74	Farm 2	Pond 1	*Li. vannamei* 2	Muscle	786	Vibrionaceae
75	Farm 2	Pond 1	*Li. vannamei* 2	Muscle	830	Shewanellaceae
76	Farm 2	Pond 1	*Li. vannamei* 2	Muscle	782	Shewanellaceae
77	Farm 2	Pond 2	Water	-	735	Bacillaceae
78	Farm 2	Pond 2	Water	-	800	Bacillaceae
79	Farm 2	Pond 2	*Li. vannamei* 1	Hepatopancreas	728	Vibrionaceae
80	Farm 2	Pond 2	*Li. vannamei* 1	Intestine	761	Bacillaceae
81	Farm 2	Pond 2	*Li. vannamei* 1	Intestine	734	Bacillaceae
82	Farm 2	Pond 2	*Li. vannamei* 1	Muscle	789	Vibrionaceae
87	Farm 3	Pond 1	*Li. vannamei 1*	Intestine	789	Bacillaceae
88a	Farm 3	Pond 2	*Li. vannamei* 1	Muscle	782	Shewanellaceae
88b	Farm 3	Pond 2	*Li. vannamei* 1	Muscle	782	Shewanellaceae
89	Farm 3	Pond 2	*Li. vannamei* 1	Muscle	816	Vibrionaceae
90	Farm 3	Pond 3	Water	-	811	Bacillaceae
91	Farm 3	Pond 3	Water	-	763	Bacillaceae
92	Farm 3	Pond 3	*Li. vannamei* 2	Intestine	844	Micrococcaceae
93a	Farm 3	Pond 3	*Li. vannamei* 2	Intestine	872	Shewanellaceae
93b	Farm 3	Pond 3	*Li. vannamei* 2	Intestine	780	Shewanellaceae
94	Farm 3	Pond 3	*Li. vannamei* 2	Intestine	734	Microbacteriaceae
95	Farm 3	Pond 3	*Li. vannamei* 2	Intestine	778	Shewanellaceae
96	Farm 3	Pond 3	*Li. vannamei* 1	Intestine	815	Shewanellaceae
98	Farm 3	Pond 4	*Li. vannamei* 2	Hepatopancreas	765	Micrococcaceae
100	Farm 3	Pond 4	*Li. vannamei* 2	Muscle	778	Aeromonadaceae
101	Farm 3	Pond 4	*Li. vannamei* 1	Intestine	807	Bacillaceae
103	Farm 3	Pond 4	*Li. vannamei* 1	Intestine	786	Micrococcaceae
105	Farm 3	Pond 3	*Li. vannamei* 2	Gill	793	Micrococcaceae
107	Farm 4	Pond 1	*Li. vannamei* 1	Intestine	781	Shewanellaceae
108	Farm 4	Pond 1	*Li. vannamei* 1	Intestine	778	Vibrionaceae
109a	Farm 4	Pond 1	*Li. vannamei* 1	Intestine	779	Aeromonadaceae
110	Farm 4	Pond 1	*Li. vannamei* 1	Intestine	787	Vibrionaceae
111	Farm 4	Pond 1	*Li. vannamei* 1	Intestine	784	Vibrionaceae
112	Farm 4	Pond 1	*Li. vannamei* 1	Intestine	786	Bacillaceae
113	Farm 4	Pond 1	*Li. vannamei* 1	Hepatopancreas	787	Vibrionaceae

114	Farm 4	Pond 1	*Li. vannamei* 1	Hepatopancreas	786	Bacillaceae
115	Farm 4	Pond 1	*Li. vannamei* 1	Hepatopancreas	787	Vibrionaceae
117	Farm 4	Pond 1	*Li. vannamei* 1	Muscle	786	Vibrionaceae
118	South China Sea	-	*Lar. polyactis* 1	Intestine	786	Vibrionaceae
119a	South China Sea	-	*Lar. polyactis* 1	Intestine	734	Vibrionaceae
119b	South China Sea	-	*Lar. polyactis* 1	Intestine	785	Vibrionaceae
120	South China Sea	-	*Lar. polyactis* 1	Intestine	726	Vibrionaceae
121	South China Sea	-	*Lar. polyactis* 1	Intestine	787	Vibrionaceae
123	South China Sea	-	*Lar. polyactis* 1	Intestine	787	Vibrionaceae
125	South China Sea	-	*Lar. polyactis* 2	Intestine	727	Vibrionaceae
126	South China Sea	-	*Lar. polyactis* 3	Intestine	788	Vibrionaceae
129	South China Sea	-	*O. rubicundus* 1	Intestine	786	Vibrionaceae
131	South China Sea	-	*O. rubicundus* 1	Intestine	725	Vibrionaceae
132	South China Sea	-	*O. rubicundus* 1	Intestine	786	Vibrionaceae
134	South China Sea	-	*O. rubicundus* 1	Intestine	785	Vibrionaceae
135	South China Sea	-	*O. rubicundus* 1	Intestine	785	Vibrionaceae
136	South China Sea	-	*O. rubicundus* 1	Intestine	781	Shewanellaceae
140	South China Sea	-	*O. rubicundus* 1	Intestine	785	Vibrionaceae
141	South China Sea	-	*O. rubicundus* 1	Intestine	788	Vibrionaceae
142	South China Sea	-	*O. rubicundus* 1	Intestine	787	Vibrionaceae
143	South China Sea	-	*O. rubicundus* 1	Intestine	786	Vibrionaceae
145	South China Sea	-	*O. rubicundus* 2	Intestine	787	Vibrionaceae
147	South China Sea	-	*O. rubicundus* 2	Intestine	786	Vibrionaceae
149a	South China Sea	-	*O. rubicundus* 2	Intestine	787	Vibrionaceae
150a	South China Sea	-	*O. rubicundus* 2	Intestine	821	Vibrionaceae
150b	South China Sea	-	*O. rubicundus* 2	Intestine	778	Vibrionaceae
151	South China Sea	-	*O. rubicundus* 2	Intestine	790	Vibrionaceae
152	South China Sea	-	*O. rubicundus* 2	Intestine	778	Vibrionaceae
154	South China Sea	-	*O. rubicundus* 2	Intestine	778	Vibrionaceae
155	South China Sea	-	*O. rubicundus* 2	Intestine	778	Shewanellaceae
155b	South China Sea	-	*O. rubicundus* 2	Intestine	783	Shewanellaceae
157	South China Sea	-	*O. rubicundus* 2	Intestine	787	Vibrionaceae
159	South China Sea	-	*O. rubicundus* 2	Intestine	782	Shewanellaceae
161a	South China Sea	-	*O. rubicundus* 2	Intestine	777	Vibrionaceae
161b	South China Sea	-	*O. rubicundus* 2	Intestine	779	Vibrionaceae
162a	South China Sea	-	*O. rubicundus* 2	Intestine	779	Vibrionaceae
163	South China Sea	-	*O. rubicundus* 2	Intestine	725	Vibrionaceae
164a	South China Sea	-	*O. rubicundus* 2	Intestine	777	Vibrionaceae
164c	South China Sea	-	*O. rubicundus* 2	Intestine	788	Bacillaceae
165a	South China Sea	-	*O. rubicundus* 2	Intestine	776	Vibrionaceae
166	South China Sea	-	*O. rubicundus* 2	Intestine	777	Vibrionaceae
167	South China Sea	-	*O. rubicundus* 2	Intestine	778	Vibrionaceae
168	South China Sea	-	*O. rubicundus* 3	Intestine	715	Vibrionaceae
173	South China Sea	-	*O. rubicundus* 3	Intestine	787	Vibrionaceae
176	South China Sea	-	*O. rubicundus* 3	Intestine	786	Vibrionaceae
180	South China Sea	-	*O. rubicundus* 3	Intestine	788	Vibrionaceae
182	South China Sea	-	*O. rubicundus* 3	Intestine	787	Vibrionaceae
184	South China Sea	-	*T. ocellatus* 1	Intestine	787	Vibrionaceae
185	South China Sea	-	*T. ocellatus* 1	Intestine	787	Vibrionaceae
186	South China Sea	-	*T. ocellatus* 1	Intestine	777	Vibrionaceae
189	South China Sea	-	*T. ocellatus* 2	Intestine	786	Vibrionaceae
192	South China Sea	-	*T. ocellatus* 2	Intestine	718	Vibrionaceae
195	South China Sea	-	*T. ocellatus* 2	Intestine	786	Vibrionaceae
196	South China Sea	-	*T. ocellatus* 2	Intestine	779	Vibrionaceae
199	South China Sea	-	*T. ocellatus* 2	Intestine	778	Vibrionaceae
200	South China Sea	-	*T. ocellatus* 2	Intestine	785	Vibrionaceae
203	South China Sea	-	*T. ocellatus* 2	Intestine	729	Vibrionaceae
206	South China Sea	-	*T. ocellatus* 2	Intestine	779	Shewanellaceae
207	South China Sea	-	*T. ocellatus* 2	Intestine	728	Paenibacillaceae
209	South China Sea	-	*T. ocellatus* 2	Intestine	779	Shewanellaceae
210	South China Sea	-	*T. ocellatus* 2	Intestine	781	Shewanellaceae
211	South China Sea	-	*T. ocellatus* 2	Intestine	789	Vibrionaceae
213	South China Sea	-	*T. ocellatus* 2	Intestine	780	Vibrionaceae
213a	South China Sea	-	*T. ocellatus* 2	Intestine	758	Brevibacteriaceae
214	South China Sea	-	*T. ocellatus* 2	Intestine	779	Vibrionaceae
215	South China Sea	-	*Pen. monodon* 2	Intestine	783	Shewanellaceae
216	South China Sea	-	*Pen. monodon* 2	Intestine	755	Micrococcaceae
218	South China Sea	-	*Pen. monodon* 2	Intestine	779	Vibrionaceae
220	South China Sea	-	*Pen. monodon* 2	Intestine	686	Vibrionaceae
221	South China Sea	-	*Pen. monodon* 3	Intestine	785	Vibrionaceae
223	South China Sea	-	*Pen. monodon* 3	Intestine	787	Vibrionaceae
224	South China Sea	-	*Pen. monodon* 3	Intestine	786	Vibrionaceae
225	South China Sea	-	*Pen. monodon* 3	Intestine	787	Vibrionaceae
226	South China Sea	-	*Pen. monodon* 3	Intestine	787	Vibrionaceae
227	South China Sea	-	*Pen. monodon* 3	Intestine	786	Vibrionaceae
228	South China Sea	-	*S. serrata* 1	Intestine	786	Vibrionaceae
229	South China Sea	-	*S. serrata* 1	Intestine	788	Vibrionaceae
230	South China Sea	-	*S. serrata* 1	Intestine	778	Vibrionaceae
231	South China Sea	-	*S. serrata* 1	Intestine	787	Vibrionaceae
232	South China Sea	-	*S. serrata* 1	Intestine	732	Vibrionaceae
233	South China Sea	-	*S. serrata* 1	Intestine	777	Vibrionaceae
234	South China Sea	-	*S. serrata* 1	Intestine	719	Vibrionaceae
235	South China Sea	-	*S. serrata* 1	Intestine	786	Vibrionaceae
236	South China Sea	-	*S. serrata* 1	Intestine	787	Vibrionaceae
237	South China Sea	-	*S. serrata* 1	Intestine	786	Vibrionaceae
238	South China Sea	-	*S. serrata* 1	Intestine	788	Vibrionaceae
239	South China Sea	-	*S. serrata* 1	Intestine	787	Vibrionaceae
240	South China Sea	-	*S. serrata* 1	Intestine	788	Vibrionaceae
241	South China Sea	-	*S. serrata* 1	Intestine	786	Vibrionaceae
242	South China Sea	-	*S. serrata* 1	Intestine	786	Vibrionaceae
243	South China Sea	-	*S. serrata* 1	Intestine	785	Vibrionaceae
244a	South China Sea	-	*S. serrata* 1	Intestine	787	Vibrionaceae
244b	South China Sea	-	*S. serrata* 1	Intestine	776	Vibrionaceae

245	South China Sea	-	*S. serrata* 1	Intestine	786	Vibrionaceae
247	South China Sea	-	*S. serrata* 1	Intestine	786	Vibrionaceae
248a	South China Sea	-	*S. serrata* 2	Intestine	788	Vibrionaceae
248b	South China Sea	-	*S. serrata* 2	Intestine	788	Vibrionaceae
249	South China Sea	-	*S. serrata* 2	Intestine	777	Vibrionaceae
250	South China Sea	-	*S. serrata* 2	Intestine	789	Vibrionaceae
251	South China Sea	-	*S. serrata* 2	Intestine	726	Vibrionaceae
252	South China Sea	-	*S. serrata* 2	Intestine	789	Vibrionaceae
253	South China Sea	-	*S. serrata* 2	Intestine	741	Vibrionaceae
254	South China Sea	-	*S. serrata* 2	Intestine	729	Vibrionaceae
255	South China Sea	-	*S. serrata* 2	Intestine	790	Bacillaceae
256	South China Sea	-	*S. serrata* 2	Intestine	714	Bacillaceae
257	South China Sea	-	*S. serrata* 2	Intestine	787	Vibrionaceae
258	South China Sea	-	*S. serrata* 2	Intestine	788	Vibrionaceae
259	South China Sea	-	*S. serrata* 2	Intestine	787	Vibrionaceae
260	South China Sea	-	*S. serrata* 2	Intestine	797	Bacillaceae
261	South China Sea	-	*S. serrata* 3	Intestine	710	Shewanellaceae

*Pond represents mud pond; tank represents cultivation tank within a recirculation aquaculture system (RAS).

a

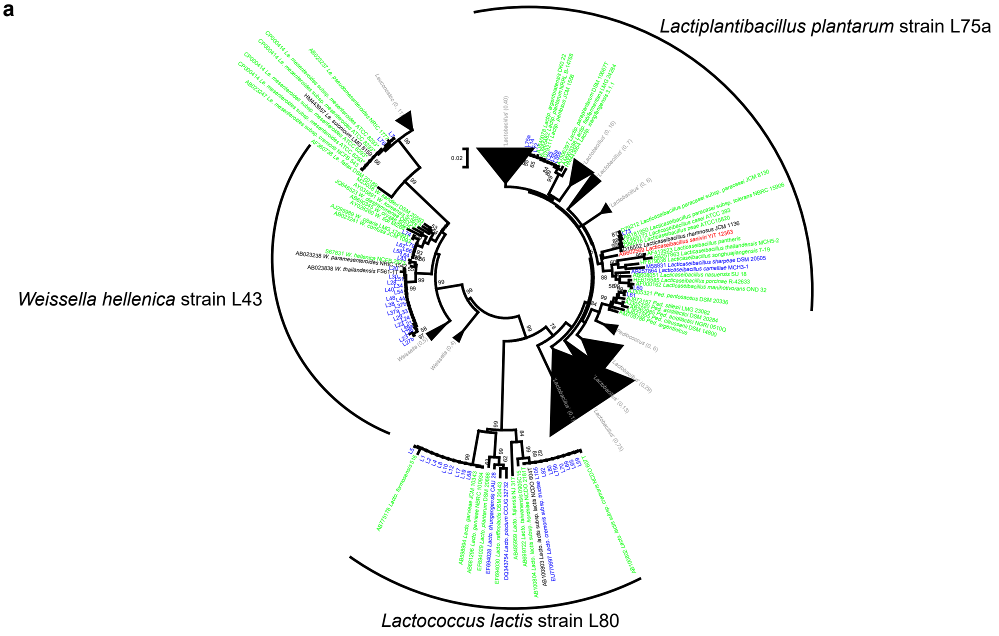

Lactiplantibacillus plantarum strain L75a
Weissella hellenica strain L43
Lactococcus lactis strain L80

b

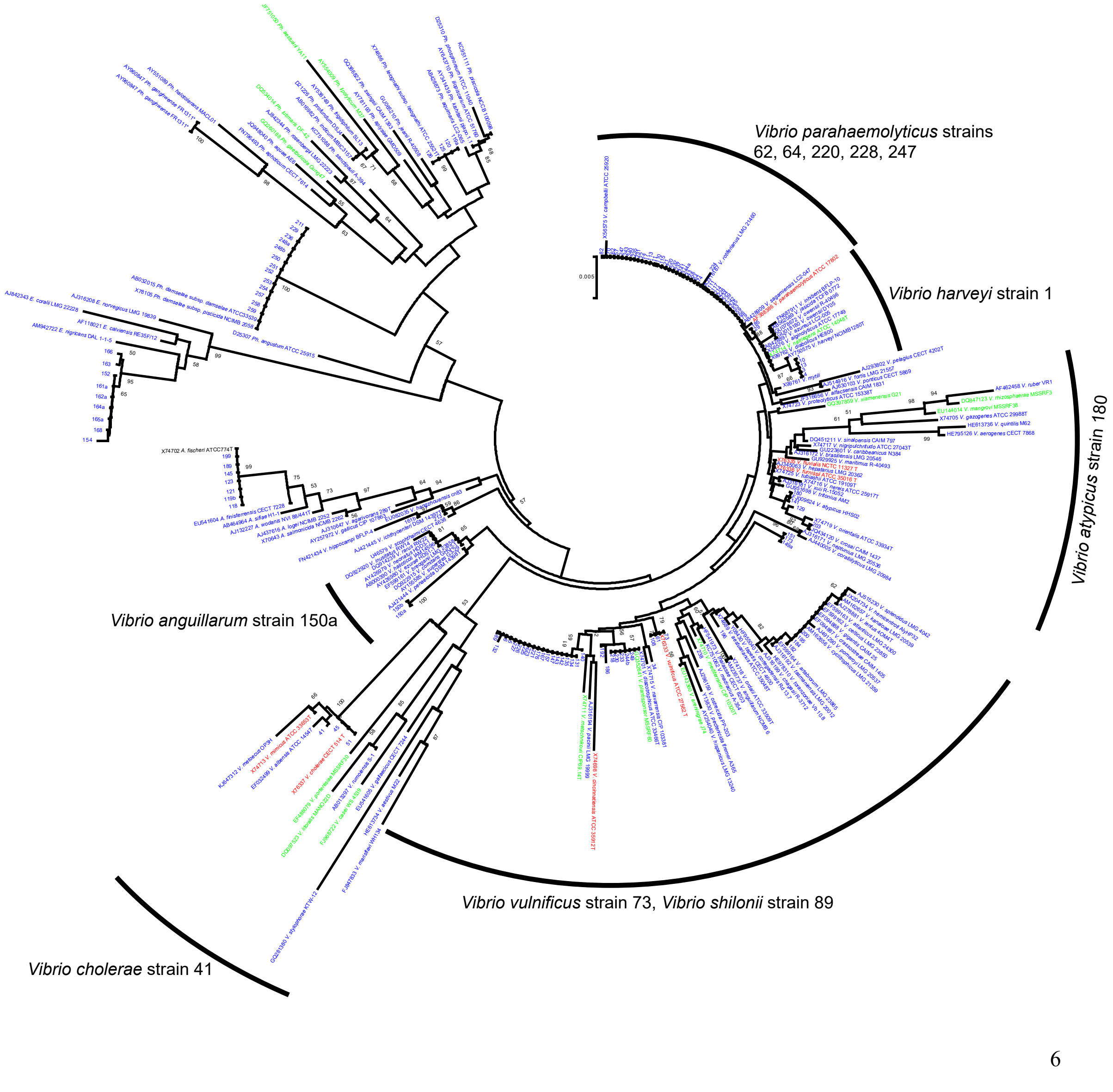

Vibrio parahaemolyticus strains 62, 64, 220, 228, 247
Vibrio harveyi strain 1
Vibrio atypicus strain 180
Vibrio anguillarum strain 150a
Vibrio vulnificus strain 73, Vibrio shilonii strain 89
Vibrio cholerae strain 41

Extended Data Fig. 1 16S rRNA gene-based phylogenetic characterization of the LAB (a) and Vibrionaceae (b) isolates. Phylogenetic trees of 16S rRNA gene sequences (≥ 1,200 bp) of 56 LAB (**a**) and 128 Vibrionaceae (**b**) isolates and their corresponding reference strains, which were 268 LAB (**a**) and 139 Vibrionaceae (**b**) type strains with good-quality sequences downloaded from the Ribosomal Database Project[4] (RDP, http://rdp.cme.msu.edu/). The analyses of the neighbour-joining[5] consensus tree were performed in Mega 7[6] using the Kimura-2-parameter method[7] to compute evolutionary distances, and the non-uniformity of evolutionary rate amongst site was modelled by the discrete Gamma distribution with Gamma parameters of 0.22 (**a**) and 0.1 (**b**). The bootstrap values indicated at the nodes are based on 1,000 bootstrap replicates[8]. Branch values lower than 50% are hidden. The scale bars indicate evolutionary distances of 0.02 (**a**) and 0.005 (**b**) nucleotide substitution per sequence position. Each strain name is preceded by the accession number. Red, blue, green and black colours indicate the strains from human-related, aquatic, terrestrial and unclear sources of isolation, respectively. The asterisks indicate the four (**a**) and two (**b**) reference strains with the same accession number possessed different RDP IDs. Each outer label displays the isolate(s) selected from the corresponding clade for the safety evaluation (**a**) or for the *in vivo* tests on *D. rerio* (**b**). (**a**) The clades distantly related to the isolates were collapsed and the first and second numbers in the parentheses following each genus name indicate the total amount of isolates and type strains in the corresponding clade, respectively. The term '*Lactobacillus*' represents the former genus name which has been reclassified into 25 genera as proposed by Zheng and colleagues[1]. (**b**) Strains from (inter)tidal area, coastal sediment, mangrove soil and salt marsh mud are marked in green.

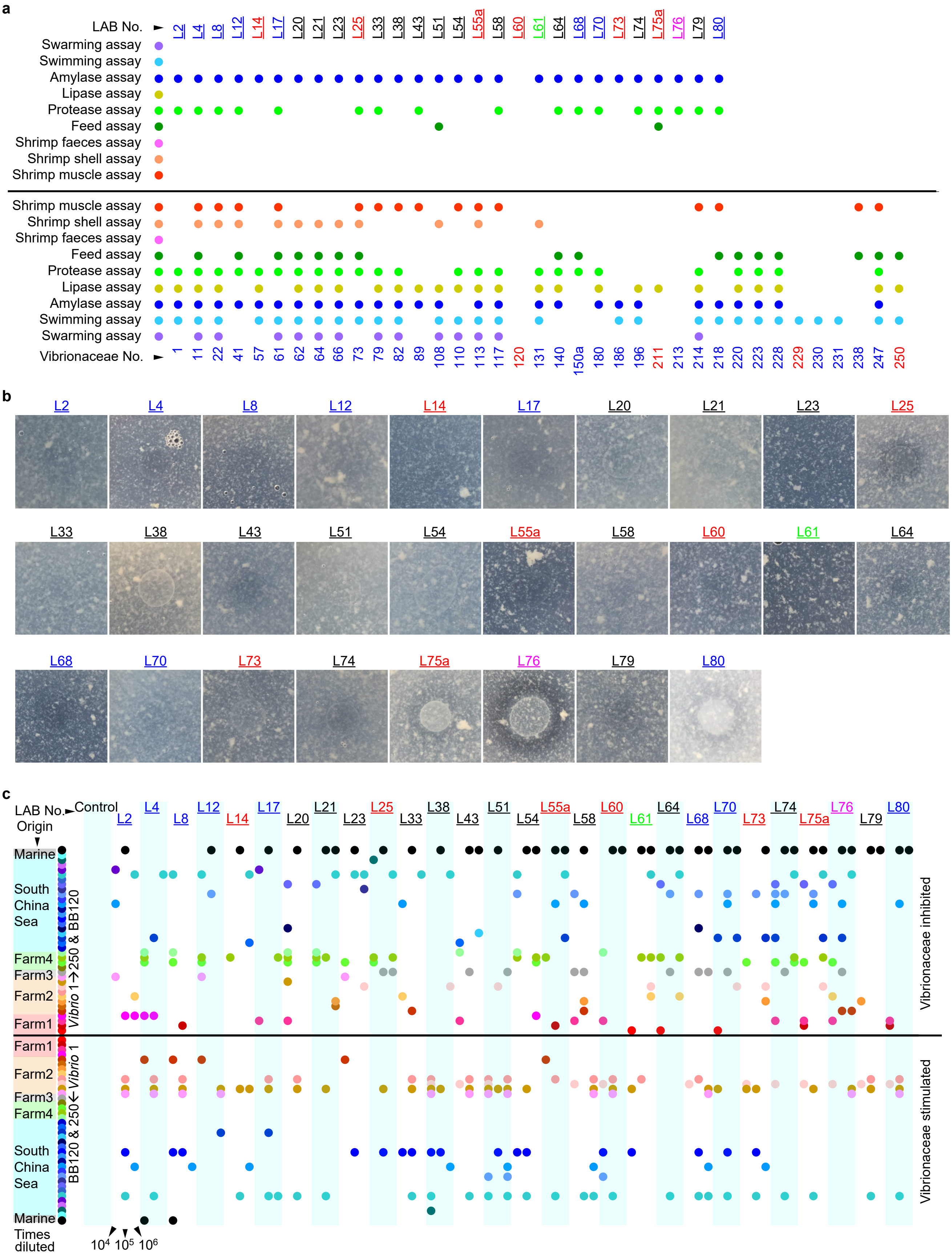
a
LAB No.
L2 L4 L8 L12 L14 L17 L20 L21 L23 L25 L33 L38 L43 L51 L54 L55a L58 L60 L61 L64 L68 L70 L73 L74 L75a L76 L79 L80
Swarming assay
Swimming assay
Amylase assay
Lipase assay
Protease assay
Feed assay
Shrimp faeces assay
Shrimp shell assay
Shrimp muscle assay
Vibrionaceae No.
1 11 22 41 57 61 62 64 66 73 79 82 89 108 110 113 117 120 131 140 150a 180 186 196 211 213 214 218 220 223 228 229 230 231 238 247 250
b
c
LAB No.
Control
Origin
Marine
South China Sea
Farm4
Farm3
Farm2
Farm1
Vibrio 1→250 & BB120
BB120 & 250←Vibrio 1
Vibrionaceae inhibited
Vibrionaceae stimulated
Times diluted
10^4 10^5 10^6

Extended Data Fig. 2 Functional characterization of the LAB and Vibrionaceae isolates. (**a**) Nutrient utilization and motility of 28 LAB and 37 Vibrionaceae isolates. Each assay is designated a unique coloured circle as indicated on the left. The presence of a filled circle indicates the isolate possessed the corresponding activity. (**b**) Protease activity of 28 LAB isolates. Each isolate was spot-inoculated on the protease indicator medium and the protease activity was determined by observation of protein degradation halo around and/or within the inoculum. (**c**) Impact of the cell-free fermented broths of the 28 LAB isolates on the proliferation of the 37 Vibrionaceae isolates. The cell-free fermented broth of each LAB isolate was diluted 10^4-, 10^5- and 10^6- times with LB10 broth and the impact of each dilution on each Vibrionaceae isolate is indicated in the order of left, middle and right of each column, respectively. '*Vibrio* 1→250 & BB120' describes the 37 Vibrionaceae isolates as listed in (**a**) in the order from isolate 1 to 250, and the type strain *V. campbellii* BB120 (black circle). Each Vibrionaceae isolate, whose origin is indicated on the left, is designated a unique coloured circle. The presence of a circle above or below the horizontal line indicates the proliferation of the Vibrionaceae isolate was consistently inhibited or stimulated by the corresponding cell-free fermented broth in the exponential growth phase within 24 hours, respectively. (**a**), (**b**) and (**c**), the strain numbers of '*Lactobacillus*' (this term represents the former genus name which has been reclassified into 25 genera as proposed by Zheng and colleagues[1]), *Lactococcus*, *Weissella*, *Pediococcus* and *Leuconostoc* isolates are underscored and marked in red, blue, black, green and pink, respectively; (**a**), the strain numbers of *Vibrio* and *Photobacterium* isolates are marked in blue and red, respectively.

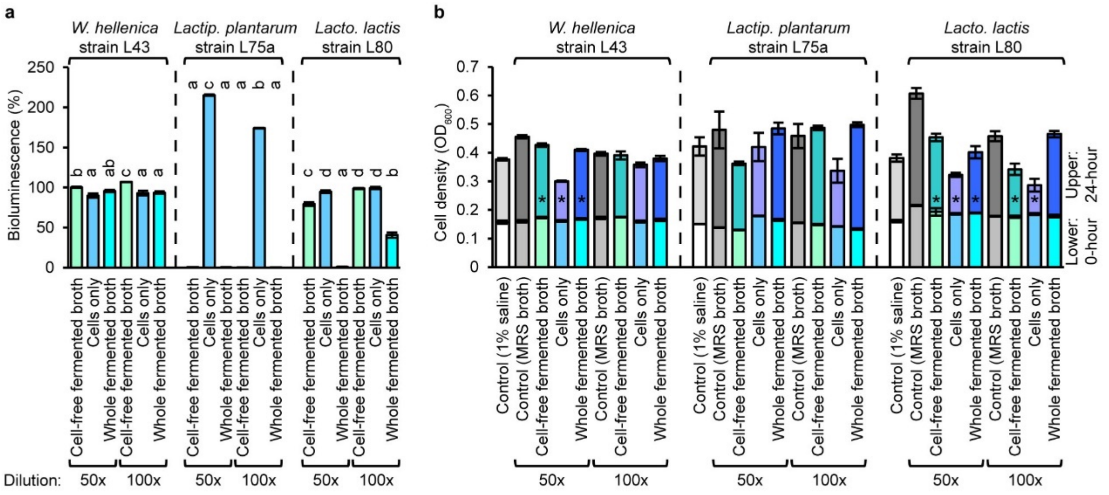

Extended Data Fig. 3 Impact of the fermented broths of *W. hellenica* strain L43, *Lactip. plantarum* strain L75a and *Lacto. lactis* strain L80 on bioluminescence (a) and proliferation (b) of the type strain *V. campbellii* BB120. *V. campbellii* BB120 was treated with 50- and 100-times-diluted cell-free supernatant, cells and whole fermented broth of each isolate, respectively. The control treatment for the cell-free and whole fermented broth was MRS broth, and for the cells of each fermented broth was 1% saline. The initial density of *V. campbellii* BB120 in each treatment was 10^8 cells ml^{-1}. The bioluminescence (**a**) of *V. campbellii* BB120 was measured within 15 min after *V. campbellii* BB120 was treated; then, the proliferation (cell density) (**b**) of *V. campbellii* BB120 was measured after 24 hours of incubation. In panel (**a**) and (**b**), the results of the three different tests (performed independently at three different times) are separated by the dotted lines. (**a**) The bioluminescence of each control treatment was set at 100% and the other corresponding treatments were normalized accordingly[9]; in the same test, treatments with different letters indicate statistically significant differences. (**b**) Each stacked bar displays the cell density in each treatment that was determined after incubation for 0 (lower stack) and 24 (upper stack) hours; amongst the OD_{600} values determined at the 24th hour in the same test, an asterisk indicates a statistically significant difference compared to the corresponding control treatment of the corresponding dilution. The error bars represent S.E.M. (*N*=3). The proliferation of *V. campbellii* BB120 was neither inhibited nor stimulated by the cell-free supernatant, the cells and the whole fermented broth of *Lactip. plantarum* strain L75a.

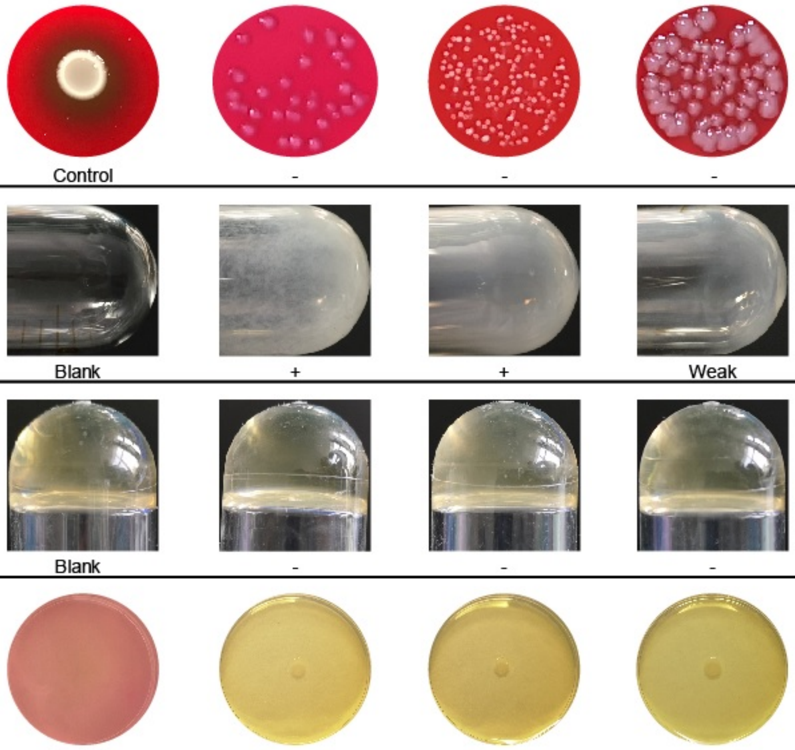

Activities		*W. hellenica* strain L43	*Lactip. plantarum* strain L75a	*Lacto. lactis* strain L80
Hemolysis (anaerobic)	Control	-	-	-
Biofilm formation	Blank	+	+	Weak
Gelatinase	Blank	-	-	-
Ammonia production (anaerobic)	Control	-	-	-
Mucin utilization		-	-	-
Cytotoxicity*	Cells only, 10^8cells ml^{-1}	Non-toxic	Non-toxic	Toxic
	Cells only, 10^7cells ml^{-1}	Non-toxic	Non-toxic	Non-toxic
	Cell-free supernatant, 10-times diluted	Toxic	Toxic	Toxic
	Cell-free supernatant, 100-times diluted	Non-toxic	Non-toxic	Non-toxic
Bile salt tolerance (Bile salt concentration)	0.1%	++	++	++
	0.5%	++	++	++
	1%	++	++	++
	1.5%	++	++	++
	2%	++	++	–
	2.5%	++	++	–
	3%	++	++	–
pH tolerance (pH value)	1	–	–	–
	2	–	–	–
	3	–	++	–
	4	++	++	–
	5	++	++	++
	6	++	++	++
	7	++	++	++

Extended Data Fig. 4 Safety evaluation of the three representative LAB isolates (I). '+' and '-' represent positive and negative for the corresponding activity, respectively. The cytotoxic activity was concluded when the EPC survival percentage of a treatment was significantly lower than that of the corresponding control treatment, based on a one-way analysis of variance and on post hoc Duncan analysis (P<0.05). '++' and '--' represent the LAB isolates were respectively capable and incapable of proliferating in the media with specific bile salt concentrations or pH values. Remarks (*): in realistic aquaculture scenarios, the applied dosage of a probiotic fermented broth is 10^4- to 10^6-times dilution, which is correspondent to the density of 10^3-10^5 cells ml^{-1}.

Extended Data Table 2 Safety evaluation of the three representative LAB isolates (II) - testing for antibiotic susceptibility. The washed cells of each isolate were resuspended in ultrapure water to reach a density of 10^9 CFU ml^{-1}. 30 µl, 10 µl and 30 µl cell suspension of *W. hellenica* strain L43, *Lactip. plantarum* strain L75a and *Lacto. lactis* strain L80 was spread-plated on each MRS agar plate (Ø6 cm), respectively. One antibiotic disc was placed at the centre of each plate, which was then incubated at 28 ℃ for 61±1 hours. The resistant (R) or sensitive (S) activity of each isolate to the corresponding antibiotic was determined based on the absence or presence of a clearing halo around the antibiotic paper disc, respectively. Each isolate was tested for each antibiotic susceptibility in triplicate.

No.	Antibiotics	Content	*W. hellenica* strain L43	*Lactip. plantarum* strain L75a	*Lacto. Lactis* strain L80
1	Penicillin	10 µg/disc	R	R	S
2	Oxacillin	1 µg/disc	R	R	S
3	Ampicillin	10 µg/disc	S	R	S
4	Carbenicillin	100 µg/disc	Activity not consistent	S	S
5	Piperacillin	100 µg/disc	S	S	S
6	Cephalexin	30 µg/disc	S	S	S
7	Cefamezin	30 µg/disc	S	S	S
8	Cefradine	30 µg/disc	S	S	S
9	Cefuroxim	30 µg/disc	R	S	S
10	Ceftazidime	30 µg/disc	S	S	S
11	Ceftriaxone	30 µg/disc	R	S	S
12	Cefoperazone	75 µg/disc	S	S	S
13	Amikacin	30 µg/disc	R	R	R
14	Gentamicin	10 µg/disc	S	R	S
15	Kanamycin	30 µg/disc	S	R	Activity not consistent
16	Neomycin	30 µg/disc	R	R	Activity not consistent
17	Tetracycline	30 µg/disc	S	S	S
18	Doxycycline	30 µg/disc	S	S	S
19	Minocycline	30 µg/disc	S	S	S
20	Erythromycin	15 µg/disc	S	S	S
21	Midecamycinum	30 µg/disc	S	S	S
22	Norfloxacin	10 µg/disc	R	R	R
23	Ofloxacin	5 µg/disc	S	S	S
24	Ciprofloxacin	5 µg/disc	S	R	S
25	Vancomycin	30 µg/disc	R	R	S
26	Polymyxin B	300 IU/disc	S	R	R
27	Compound Sulfamethoxazole	Sulfamethoxazole 23.75 µg/disc Trimethoprim 1.25 µg/disc	R	S	S
28	Furazolidone	100 µg/disc	R	S	S
29	Chloramphenicol	30 µg/disc	S	S	S
30	Clindamycin	2 µg/disc	R	S	S
31	Roxithromycin	15 µg/disc	S	S	S
32	Enrofloxacin	10 µg/disc	S	S	S
33	Florfenicol	30 µg/disc	S	S	S
34	Cefixime	5 µg/disc	S	S	S
35	Lincomycin	2 µg/disc	R	R	S
36	Aztreonam	30 µg/disc	R	R	R
37	Cefoperazone-Sulbactam	Cefoperazone sodium 75 µg/disc Sulbactam 75 µg/disc	S	S	S
38	Sultamicillin	Sulbactam 10 µg/disc Ampicillin 10 µg/disc	S	S	S
39	Metronidazole	5 µg/disc	R	R	S
40	Cefotaxime	30 µg/disc	S	S	S
41	Augmentin	Amoxicillin: 20 µg/disc Clavulanic acid: 10 µg/disc	S	S	S
42	Bacitracin	0.04 U/disc	R	R	R
43	Acetylspiramycin	30 µg/disc	S	S	S
44	Cefepime	30 µg/disc	R	R	S
45	Tobramycin	10 µg/disc	S	R	R
46	Spectinomycin	100 µg/disc	R	S	R
47	Gatifloxacin	5 µg/disc	S	S	S

48	Furadantin	300 μg/disc	R	S	S
49	Streptomycin	10 μg/disc	R	R	R
50	Azithromycin	15 μg/disc	S	S	S
51	Clarithromycin	15 μg/disc	S	S	S
52	Cefoxitin	30 μg/disc	S	S	S
53	Fleroxacin	5 μg/disc	R	S	S
54	Furazolidone	100 μg/disc	R	S	S
55	Fosfomycin	200 μg/disc	R	R	S
56	Cephalothin	30 μg/disc	S	S	S
57	Novobiocin	30 μg/disc	S	S	S
58	Rifampicin	5 μg/disc	S	S	R
59	Levofloxacin	5 μg/disc	S	R	R
60	Imipenem*	10 μg/disc	S	S	S
61	Methoxillin*	75 μg/disc	S	S	S
62	Tazobactam*	110 μg/disc	S	S	S
63	Teicoplanin*	30 μg/disc	R	R	S
64	Meropenem*	10 μg/disc	S	S	S
65	Nudic acid*	30 μg/disc	R	R	R
66	Amoxicillin	20 μg/disc	S	S	S
67	Nystatin dihydrate	100 μg/disc	R	R	R
68	Optochin**	5 μg/disc	not determined	R	R
69	Sulfafurazole	300 μg/disc	S	S	S

Remarks: Manufacturers for antibiotics that are marked with * and ** are Changde BKMAM Biotechnology Co., Ltd. (Changde, Hunan, China) and Hangzhou Binhe Microorganism Reagent Co., Ltd. (Hangzhou, Zhejiang, China), respectively; manufacturer for the other antibiotics is Hangzhou Microbial Reagent Co., Ltd. (Hangzhou, Zhejiang, China).

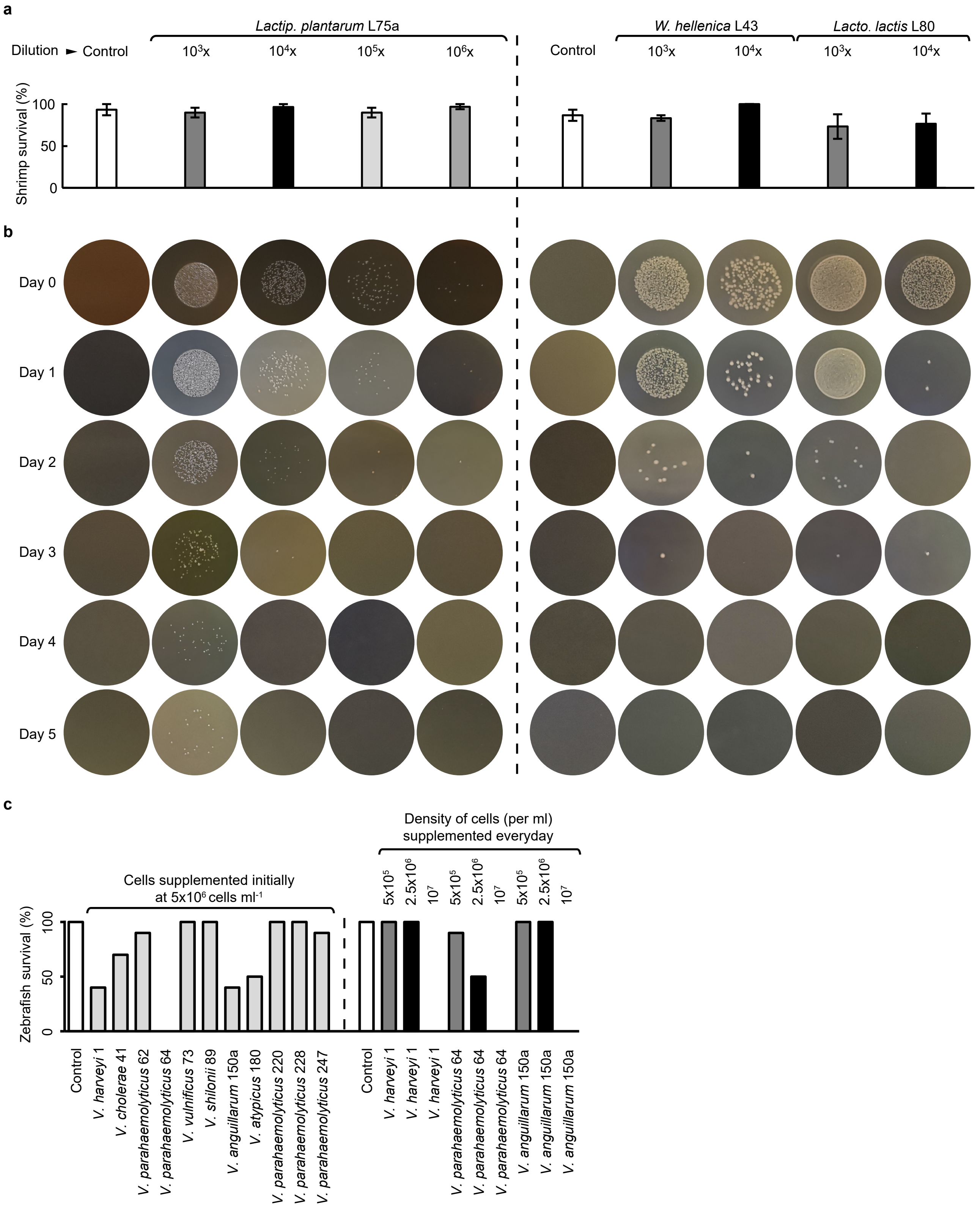
a
Lactip. plantarum L75a
W. hellenica L43
Lacto. lactis L80
Dilution ► Control
10³x
10⁴x
10⁵x
10⁶x
Control
Shrimp survival (%)
100
50
0
b
Day 0
Day 1
Day 2
Day 3
Day 4
Day 5
c
Cells supplemented initially at 5x10⁶ cells ml⁻¹
Density of cells (per ml) supplemented everyday
5x10⁵
2.5x10⁶
10⁷
Zebrafish survival (%)
Control
V. harveyi 1
V. cholerae 41
V. parahaemolyticus 62
V. parahaemolyticus 64
V. vulnificus 73
V. shilonii 89
V. anguillarum 150a
V. atypicus 180
V. parahaemolyticus 220
V. parahaemolyticus 228
V. parahaemolyticus 247

Extended Data Fig. 5 Safety evaluation of the three representative LAB isolates (III) - pathogenicity to *Li. vannamei* ((a) and (b)), and preliminary tests to screen for the most virulent *Vibrio* isolate and its pathogenic dose on *D. rerio* (c). (**a**) and (**b**), on day 0, the rearing water of *Li. vannamei* was supplemented with 10^3-, 10^4-, 10^5- and 10^6-times-diluted fermented broth of *Lactip. plantarum* strain L75a, and 10^3- and 10^4-times-diluted fermented broths of *W. hellenica* strain L43 and *Lacto. Lactis* strain L80. Each treatment was conducted in triplicate. *Li. vannamei* in the control treatments were untreated. (**a**) The mean survival percentage of *Li. vannamei* after 5 days of cultivation. The error bars represent the S.E.M. (N=3). (**b**) The LAB cell density in the rearing water of each replicate of each treatment was determined everyday by spot-inoculation of 5 µl rearing water in triplicate on MRS agar. (**c**) The *D. rerio* were treated with i) 11 *Vibrio* isolates, representing those from the six biggest phylogenetic clades of *Vibrio* species, at the initial density of 5×10^6 cells ml^{-1} for 7 days (left, the *Vibrio* cells were added to the rearing water only on day 0); ii) the 3 most virulent *Vibrio* isolates, which were tested in the former *D. rerio* bioassay (left), at the density of 5×10^5, 2.5×10^6 and 10^7 cells ml^{-1} for 15 days (right, the *Vibrio* cells were added to the rearing water everyday). Each treatment was conducted in one replicate. The survival percentage of *D. rerio* was determined at the end of each test. The fish in the control treatments were untreated. The results of the two different tests (performed independently at two different times) in the same panel are separated by the dotted line.

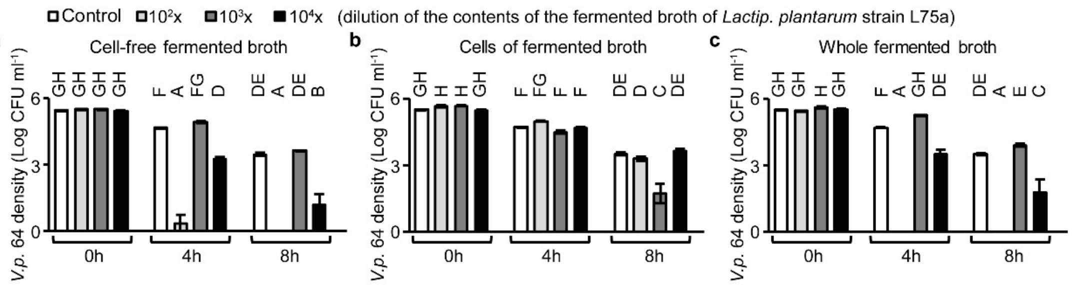

Extended Data Fig. 6 Impact of the cell-free supernatant (a), the cells (b) and the whole fermented broth (c) of *Lactip. plantarum* strain L75a on the proliferation of *V. parahaemolyticus* strain 64 (*V.p.* 64). Each content of the fermented broth was diluted 10^2-, 10^3- or 10^4-times with 0.5% saline. The washed cells of *V. parahaemolyticus* strain 64 were added to each diluted content to an initial density of 10^5 cells ml^{-1}. Each treatment was performed in triplicate. After coincubation for 0, 4 and 8 hours, the colony forming unit (CFU) of the *Vibrio* in each treatment was determined on TCBS agar. The untreated *Vibrio* cells (control treatment) were suspended in 0.5% saline. The error bars represent the S.E.M. (*N*=3). Different capital letters indicate statistically significant differences. In the treatments with only cells and whole fermented broth of *Lactip. plantarum* strain L75a, the CFU of *Lactip. plantarum* strain L75a was determined on MRS agar in triplicate; the results showed that the cells of *Lactip. plantarum* strain L75a in each corresponding treatment was consistent during the 8 hours (data not shown).

a

	Lactip. plantarum strain L75a	*V. parahaemolyticus* strain 64
Sequencing technology	PacBio Sequel I	PacBio Sequel I
<u>Assembly statistics</u>		
Genome size (Mb)	3.21325	5.1854
# scaffolds	5	4
# contigs	5	4
# reads	876,112	1,038,178
Mean subread length (bp)	13,342.97	16,518.5
N50 subread length (bp)	21,315	25,932
GC%	44.36%	45.25%
<u>Genomic features</u>		
# genes	5,074	7,996
# coding DNA sequences (CDSs)	4,993	7,830
# hypothetical proteins	2,651	4,058
# rRNA genes	16	34
# tRNA genes	64	131
# tmRNA	1	1

b

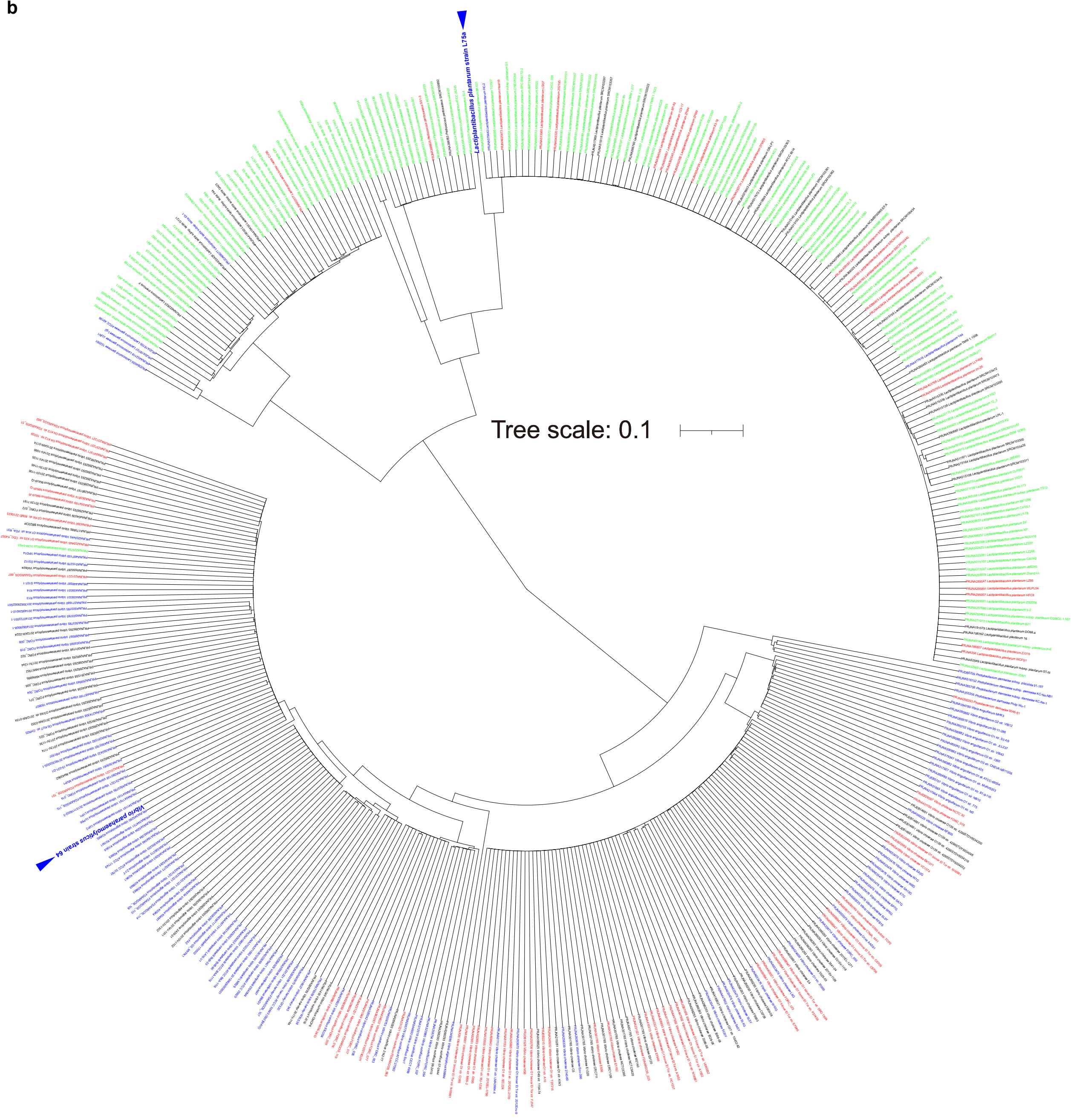

Extended Data Fig. 7 Assembly statistics and features of the genome sequences of *Lactip. plantarum* strain L75a and *V. parahaemolyticus* strain 64 (a), and whole genome-based phylogenetic characterization of these two isolates (b). (**b**) Genome sequences of the reference strains, including 191 LAB and 225 Vibrionaceae strains, with complete level as shown in GenBank were downloaded from RefSeq database[10]. The phylogenetic tree of the orthogroups of *Lactip. plantarum* strain L75a, *V. parahaemolyticus* strain 64 and the 416 reference strains was constructed with STAG in OrthoFinder[11] and visualized with iTOL (https://itol.embl.de/)[12]; default settings were applied in both tools. Each strain name is preceded by the BioProject accession number of GenBank. Red, blue, green and black colours indicate the strains from human-related, aquatic, terrestrial and unclear sources of isolation, respectively. The scale bar indicates 0.1 amino acid substitutions per site.

Extended Data Table 3 Assessment of exploitation of carbon sources by *Lactip. plantarum* strain L75a and *V. parahaemolyticus* strain 64. The cells of these two isolates were washed three times and resuspended respectively in ultrapure water and 1% saline to obtain the density of 10^6 cells ml^{-1}. 130 μl of each cell suspension was added into each well of a Biolog EcoPlate™. The plates were incubated at 28 °C and determined for the OD_{590} values after 5 days of incubation. '+' and '-' represent the isolates were able and unable to proliferate in the corresponding carbon source in the testing duration, respectively.

Carbon source	Exploitation of carbon sources	
	Lactip. plantarum strain L75a	*V. parahaemolyticus* strain 64
Water	-	-
β-Methyl-D-Glucoside	-	+
D-Galactonic Acid γ-Lactone	-	-
L-Arginine	-	+
Pyruvic Acid Methyl Ester	-	+
D-Xylose	-	-
D-Galacturonic Acid	-	-
L-Asparagine	-	+
Tween 40	-	+
i-Erythritol	-	+
2-Hydroxy Benzoic Acid	-	-
L-Phenylalanine	-	-
Tween 80	-	+
D-Mannitol	-	+
4-Hydroxy Benzoic Acid	-	-
L-Serine	-	+
α-Cyclodextrin	-	+
N-Acetyl-D-Glucosamine	-	+
γ-Hydroxybutyric Acid	-	+
L-Threonine	-	+
Glycogen	-	+
D-Glucosaminic Acid	-	-
Itaconic Acid	-	-
Glycyl-L-Glutamic Acid	-	+
D-Cellobiose	-	-
Glucose-1- Phosphate	-	+
α-Ketobutyric Acid	-	-
Phenylethylamine	-	-
α-D-Lactose	-	-
D,L-α-Glycerol Phosphate	-	+
D-Malic Acid	-	-
Putrescine	-	+

References

1 Zheng, J. *et al.* A taxonomic note on the genus Lactobacillus: Description of 23 novel genera, emended description of the genus Lactobacillus Beijerinck 1901, and union of Lactobacillaceae and Leuconostocaceae. *International Journal of Systematic and Evolutionary Microbiology* **70**, 2782-2858, doi:10.1099/ijsem.0.004107 (2020).

2 FAO & WHO. Guidelines for the Evaluation of Probiotics in Food. 11 (FAO/WHO, London Ontario, Canada 2002).

3 Altschul, S. F., Gish, W., Miller, W., Myers, E. W. & Lipman, D. J. Basic local alignment search tool. *Journal of molecular biology* **215**, 403-410, doi:10.1016/s0022-2836(05)80360-2 (1990).

4 Cole, J. R. *et al.* Ribosomal Database Project: data and tools for high throughput rRNA analysis. *Nucleic Acids Research* **42**, D633-D642, doi:10.1093/nar/gkt1244 (2013).

5 Saitou, N. & Nei, M. The neighbor-joining method: a new method for reconstructing phylogenetic trees. *Molecular biology and evolution* **4**, 406-425, doi:10.1093/oxfordjournals.molbev.a040454 (1987).

6 Kumar, S., Stecher, G. & Tamura, K. MEGA7: Molecular Evolutionary Genetics Analysis Version 7.0 for Bigger Datasets. *Molecular Biology and Evolution* **33**, 1870-1874, doi:10.1093/molbev/msw054 (2016).

7 Kimura, M. A simple method for estimating evolutionary rates of base substitutions through comparative studies of nucleotide sequences. *Journal of molecular evolution* **16**, 111-120, doi:10.1007/bf01731581 (1980).

8 Felsenstein, J. Confidence limits on phylogenies: an approach using the bootstrap. *Evolution; international journal of organic evolution* **39**, 783-791, doi:10.1111/j.1558-5646.1985.tb00420.x (1985).

9 Yang, Q. *et al.* Indole signalling and (micro)algal auxins decrease the virulence of Vibrio campbellii, a major pathogen of aquatic organisms. *Environmental microbiology* **19**, 1987-2004, doi:10.1111/1462-2920.13714 (2017).

10 Pruitt, K. D. *et al.* RefSeq: an update on mammalian reference sequences. *Nucleic Acids Research* **42**, D756-D763, doi:10.1093/nar/gkt1114 (2013).

11 Emms, D. M. & Kelly, S. OrthoFinder: phylogenetic orthology inference for comparative genomics. *Genome Biology* **20**, 238, doi:10.1186/s13059-019-1832-y (2019).

12 Letunic, I. & Bork, P. Interactive Tree Of Life (iTOL) v4: recent updates and new developments. *Nucleic Acids Research* **47**, W256-W259, doi:10.1093/nar/gkz239 (2019).
